# Radiomics for glioblastoma survival analysis in pre-operative MRI: exploring feature robustness, class boundaries, and machine learning techniques

**DOI:** 10.1186/s40644-020-00329-8

**Published:** 2020-08-05

**Authors:** Yannick Suter, Urspeter Knecht, Mariana Alão, Waldo Valenzuela, Ekkehard Hewer, Philippe Schucht, Roland Wiest, Mauricio Reyes

**Affiliations:** 1 ARTORG Center for Biomedical Engineering Research, University of Bern, Bern, Switzerland; 2 Insel Data Science Center, Inselspital, Bern University Hospital, Murtenstrasse 42, CH-3008 Bern, Switzerland; 3 Radiology Department, Spital Emmental, Burgdorf and Langnau, Switzerland; 4 Departamento de Eletrónica Industrial, University of Minho, Braga, Portugal; 5 Institute of Pathology, University of Bern, Bern, Switzerland; 6 Department of Neurosurgery, Inselspital, Bern University Hospital, Bern, Switzerland; 7 Support Center for Advanced Neuroimaging, Inselspital, Bern University Hospital, Bern, Switzerland

**Keywords:** Glioblastoma multiforme, MRI radiomics, Overall survival classification, Multi-center, Robustness

## Abstract

**Background:**

This study aims to identify robust radiomic features for Magnetic Resonance Imaging (MRI), assess feature selection and machine learning methods for overall survival classification of Glioblastoma multiforme patients, and to robustify models trained on single-center data when applied to multi-center data.

**Methods:**

Tumor regions were automatically segmented on MRI data, and 8327 radiomic features extracted from these regions. Single-center data was perturbed to assess radiomic feature robustness, with over 16 million tests of typical perturbations. Robust features were selected based on the Intraclass Correlation Coefficient to measure agreement across perturbations. Feature selectors and machine learning methods were compared to classify overall survival. Models trained on single-center data (63 patients) were tested on multi-center data (76 patients). Priors using feature robustness and clinical knowledge were evaluated.

**Results:**

We observed a very large performance drop when applying models trained on single-center on unseen multi-center data, e.g. a decrease of the area under the receiver operating curve (AUC) of 0.56 for the overall survival classification boundary at 1 year. By using robust features alongside priors for two overall survival classes, the AUC drop could be reduced by 21.2%. In contrast, sensitivity was 12.19% lower when applying a prior.

**Conclusions:**

Our experiments show that it is possible to attain improved levels of robustness and accuracy when models need to be applied to unseen multi-center data. The performance on multi-center data of models trained on single-center data can be increased by using robust features and introducing prior knowledge. For successful model robustification, tailoring perturbations for robustness testing to the target dataset is key.

## Background

During the past decade, pattern recognition of medical imaging data has been successfully applied to a wide range of disease types. Publicly available datasets, especially in oncology, such as the cancer imaging archive (TCIA) [1], have been vital for this development, to enable machine learning (ML) researchers and clinicians to investigate imaging features and perform radiomics analyses [2]. Notable examples of radiomics based on imaging studies in oncology include treatment outcome prediction, lung cancer phenotyping, and identifying pseudo-progresson in patients with Glioblastoma multiforme (GBM) [3–5]. Most studies focus on the accuracy of predictive models on a given dataset. However, next to accuracy, we postulate that robustness of imaging features to factors such as variability in imaging protocols, different vendors, inter-rater tumor segmentation variability, patient motion, and overall image quality is fundamental for a successful translation of these technologies to the clinical workflow.

Several factors negatively affect the robustness of imaging features. These include differences in imaging protocols across vendors, image reconstruction processes, and image quality (e.g., [6, 7]). However, since multi-center data is not readily available, new ML models are usually developed and tested on single-center data where such factors are not observed. We note that ML includes the branch of Deep Learning. Hence, in order to assess the robustness of imaging features for real-world scenarios, we propose to simulate the variability of imaging parameters, as seen in multi-center datasets. In the following, we refer to such simulated variability as perturbations, which are designed based on recommended imaging protocols for GBM patients [8].

GBM is the most frequent primary brain tumor in humans and ranks highest on the World Health Organization’s grading scheme [9]. Due to its rapid growth and infiltrative nature, the median overall survival is only 14 months. The current standard-of-care is maximum safe resection, followed by chemo- and radiotherapy [10].

In this study, we propose and make available (Code at https://github.com/ysuter/gbm-robustradiomics), a feature robustness analysis pipeline to analyze the robustness of radiomic features derived from multisequence MRI for the task of overall survival (OS) prediction of GBM patients. We leverage state of the art in OS prediction from the Brain Tumor Segmentation Challenge (BraTS), which since 2017 includes a sub-challenge for OS prediction [11], as well as other published body of work, e.g., [12–16] presenting a variety of metrics, class boundaries and validation schemes. Due to the inter-relation among imaging features, feature selection methods, and prediction models, we perform a high-throughput benchmark analysis, utilizing a single and multi-center dataset, along with a scheme to simulate common perturbations such as variability of the imaging protocol, inter-rater tumor segmentation variability (from where imaging features are typically derived), and k-space undersampling employed for faster image reconstruction.

**Fig. 1:**
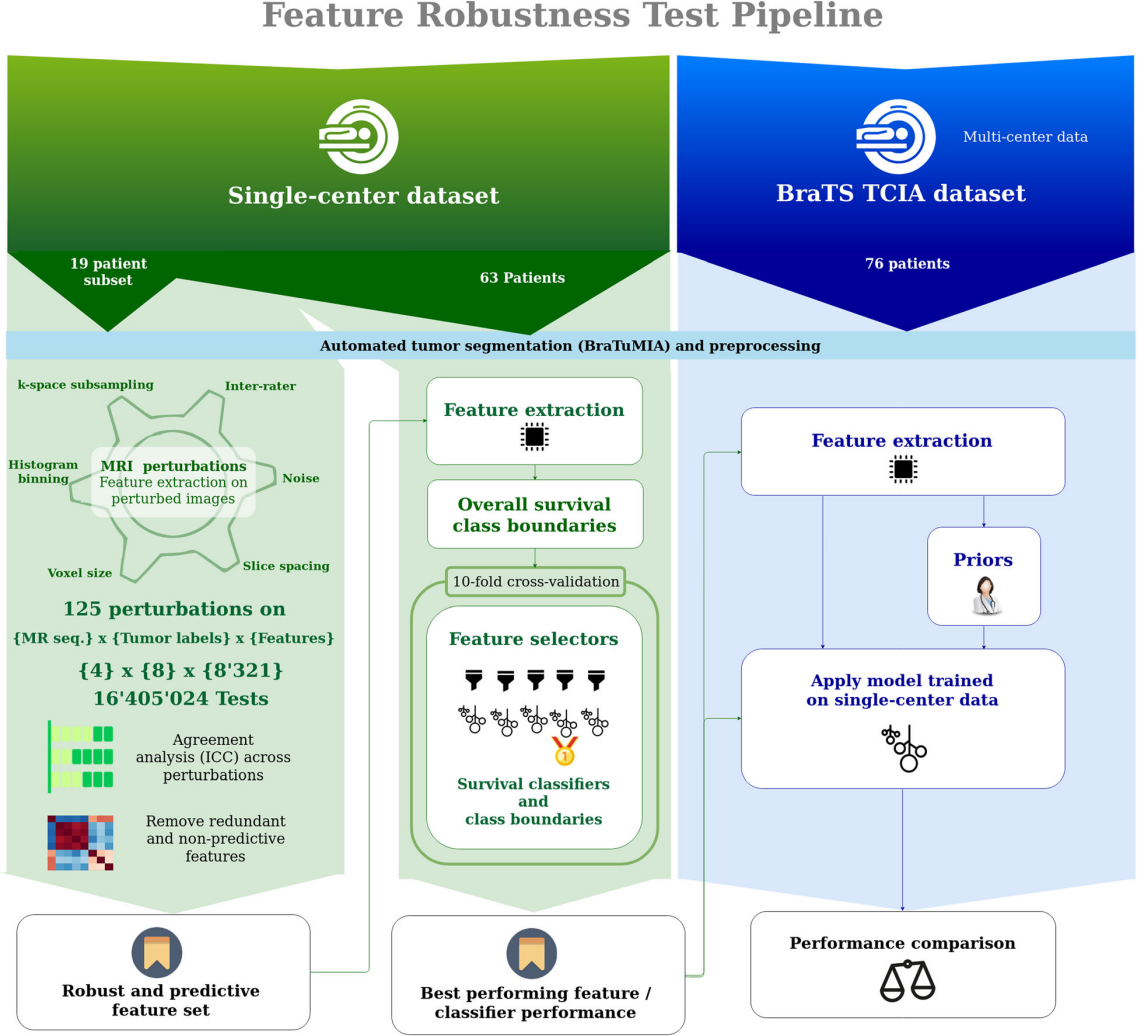
Pipeline for the proposed radiomic feature robustness assessment. A set of single-center MRI images is selected. After pre-processing and automated tumor segmentation, the images are artificially perturbed. For each perturbation type, the robustness is metered by the intraclass correlation coefficient (ICC(2,1)). Measuring agreement and not only consistency of underlying features is key for transferring trained machine learning (ML) models to a different dataset. Redundant features are removed from the robust features. Subsequently, combinations of feature selectors and ML models are tested on different survival class boundaries. The best performing model is tested on a multi-center dataset (TCIA subset of BraTS)

Our study setup consists of three parts: (a) feature robustness analysis on single-center data, (b) analysis of feature selector and machine learning techniques using robust features, and (c) multicenter performance analysis of OS prediction using the found combination of chosen robust features and ML model, and integrated clinical prior knowledge. Figure 1 shows an overview of the experimental setup.

In the next sections, we describe the main components of the proposed robustness analysis approach, single and multi-center data used for evaluation, as well as our main findings.

## Materials and methods

### Data

#### Single-center data

The records of 91 patients with newly diagnosed GBM who underwent preoperative MRI between August 2008 and December 2013 and treated with resection and temozolomide-based chemoradiation [17] were reviewed retrospectively. Patient inclusion criteria were 1) pathologically confirmed primary GBM, 2) known OS, 3) pre-operative MRI with postcontrast T1-weighted (T1c), T1-weighted (T1), T2-weighted (T2), and T2 fluid-attenuated inversion recovery (FLAIR) images. Patients with unknown OS time (*n* = 3) and missing or low-quality pre-operative MRI sequences (*n* = 25) were excluded, with a remaining study population of 63 patients (mean age: 62.75 years, standard deviation 9.96 years, mean OS: 22.77 months, standard deviation: 14.74 months). We selected a subset of 19 patients with homogeneous acquisition parameters for robustness testing. After robustness testing, the full dataset with 63 patients was used to assess the performance of feature selectors and ML model combinations.

The ethics committee approved the study and waived written informed consent.

#### Public multi-center data (BraTS TCIA)

To date, the only publicly available high-grade glioma dataset with survival information is the BraTS dataset [18], which consists of pre-treatment MRI data with patient age, survival, and extent-of-resection information. Due to our interest in the acquisition parameters, we consider the subset originating from the TCIA database [1, 19], where this information is available. The data for the survival prediction task includes MRI data from seven different centers, two different vendors, and eight MRI models, comprising 76 patients (mean age: 59.46 years, standard deviation: 13.19 years, mean OS: 14.78 months, standard deviation: 11.98 months). These images have already been skull-stripped, resampled to 1 mm voxel size, and all MRI sequences co-registered to the T1c sequence, according to [18].

The OS and age distributions of both datasets are visualized in the supplementary material, Figure S1.

#### Pre-processing and automated tumor segmentation

All single-center images were skull-stripped and resampled to match the BraTS data. Automated tumor segmentation was performed using BraTuMIA [20, 21].

BraTuMIA outputs labels for contrast-enhancement, necrosis, non-enhancing tumor, and edema. Since previous studies use different tumor labels (e.g., [13]). We combined the four labels to yield eight single and combined labels: contrast-enhancement (cet), non-enhancing tumor (net), necrosis (nec), edema (ed), whole tumor (wt, all labels combined), core (all labels except edema), necrosis and non-enhancement combined (net_ncr), and non-enhancement combined with the edema (net_ed).

### Class boundaries for OS survival prediction

Previous studies use a variety of OS class boundary definitions: Either a data-driven boundary defined by the distribution on a given dataset, or a clinically-motivated definition based on the median survival. Accordingly, we tested classification into two and three OS classes to ensure comparability to previous research.

To keep the analysis concise, we report the experiments for three OS classes in the supplementary material.

To test classification into two OS classes, we tested four different class boundaries: 304.2 days (10 months), 365 days (1 year), 425.8 days (15 months), and 540 (18 months). The 10 and 18 months class boundaries are used in the BraTS OS prediction challenge [11], and the 1 and 2 year OS is often reported in risk stratification studies and clincal reports (e.g., [10, 13])

### Radiomic features

We selected imaging features that cover widely applied types in previous studies. We analyzed all 120 features provided by PyRadiomics [22], extracted on the pre-processed MRI images. It includes shape (*n* = 26), first-order (*n* = 19), gray level co-occurrence matrix (GLCM, *n* = 24), gray level size zone matrix (GLSZM, *n* = 16), gray level run length matrix (GLRLM, *n* = 16), neighborhood gray-tone difference matrix (NGTDM, *n* = 5), and gray level dependence matrix (GLDM, *n* = 14) features [22].

Tumor location is known to affect the survival time of patients (e.g., [23]). In order to include this information, we registered each case to an atlas image [24], and computed the centroids for each segmentation label, resulting in *n* = 8 features per case.

End-to-end deep learning (DL) has been attempted for OS prediction in patients with GBM but showed unstable results [25]. We included *deep features* proposed by Lao et al. [13], where a convolutional neural network (CNN) pre-trained on the ILSVRC-2012 dataset [26] is used to extract features from the two fully-connected layers, resulting in *n* = 8192 deep features.

The last feature type considered in our study characterizes the shape of the contrast-enhancing tumor. Pérez-Beteta et al. [15] demonstrated the predictive performance of pre-treatment tumor geometry. This class of shape features (*n* = 7) is hereafter referred to as enhancement geometry.

All previously described radiomic features were extracted from all four MRI sequences and all eight segmentation labels.

### Feature robustness

We evaluated a wide range of perturbations that affect the MRI image quality to an extent expected in a multi-center setting. To define the range of perturbations, we rely on the imaging guidelines in [8] and visual inspection by a neuroradiologist:

- Voxel size and axial slice spacing, with variations generated according to a reference MRI imaging protocol, as presented in [8] for GBM patients.
- K-space subsampling: Randomly masking the image in the frequency domain using 80 to 100% of the k-space information, with the range selected by visual assessment.
- Inter-rater manual segmentation variability: Elastic deformation of all labels, such that the inter-rater Dice coefficient [27] matches the reported variability in [18] (supplementary material, Table S1 and Figure S2).
- Additive Gaussian noise, with its level set such that the signal-to-noise ratio (SNR) does not exceed the mean SNR of the single-center data plus one standard deviation (supplementary material, Figure S3).
- Quantization / binning of gray values: High-order radiomics features require histogram quantization/binning. We varied the bin width for higher-order PyRadiomics features within the recommended range in the PyRadiomics package documentation. Since consistent binning is straightforward in an image processing pipeline, no feature was excluded based on this perturbation.

**Fig. 2:**
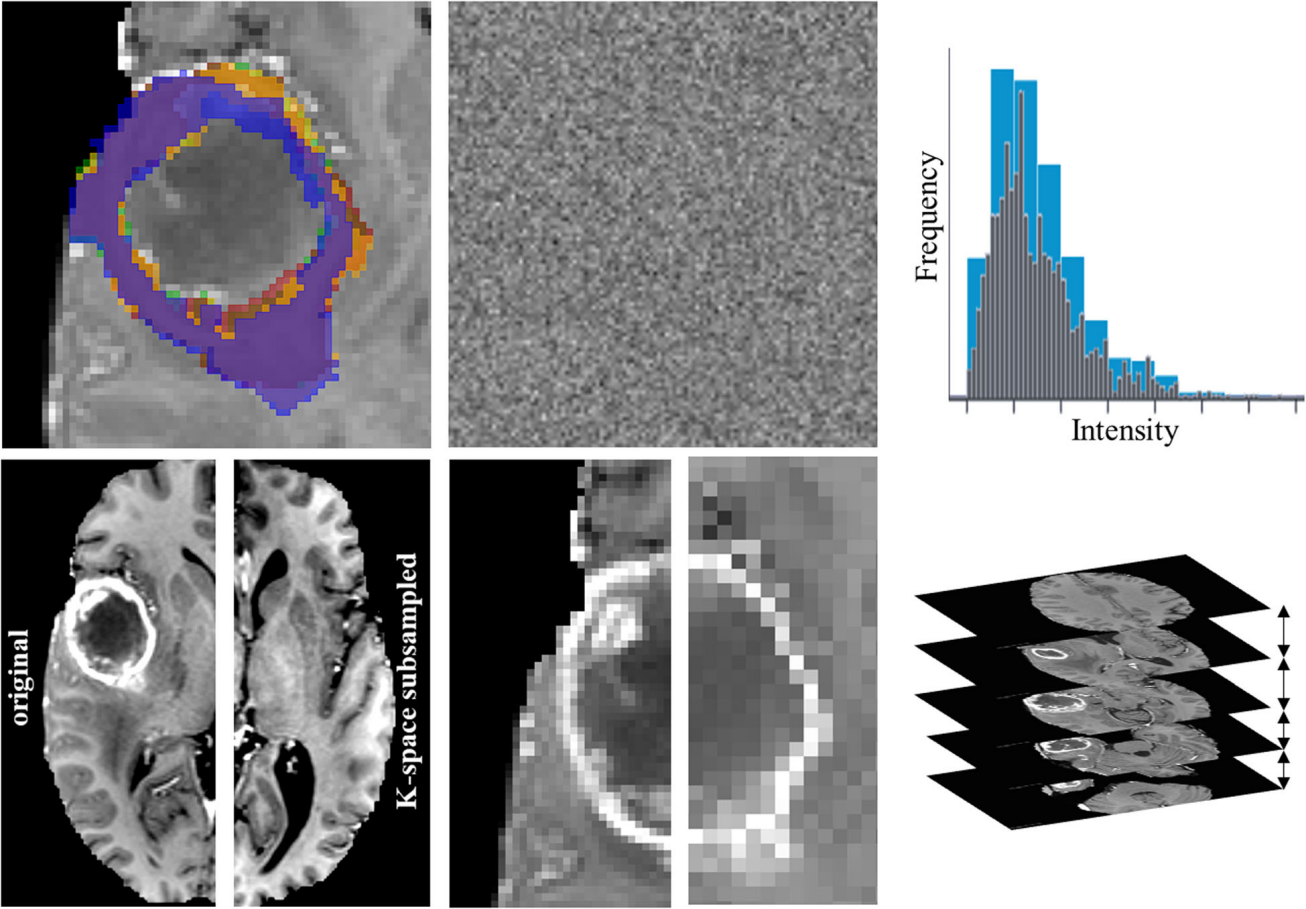
Perturbations applied to the single-center data to simulate expected multi-center data quality. Top row: left: Inter-rater simulation by deforming the labels from the automated tumor segmentation; middle: Additive Gaussian noise to match SNR range, measured on a healthy white matter segmentation; right: Adjusting the bin width within the range in the PyRadiomics documentation. Bottom row: K-space subsampling (left original, right subsampled, contrast increased for both for visualization); middle: Voxel size changed isotropically; right: Variations in axial slice spacing

These perturbations are visualized in Fig. 2 and detailed in Table 1.To ensure reproducibility, we provide all PyRadiomics feature extraction settings files and Python code used to generate perturbations (https://github.com/ysuter/gbm-robustradiomics).

**Table 1:** Overview of perturbations applied to simulate multi-center data. Perturbation types and ranges vary with labels and MRI sequences according to the recommendation in [8]

Perturbation	Sequences	Segmentaion Labels	Perturbation assessment or range	Number of perturbations per sequence/label
Additivie Gaussian noise	All	–	Match SNR range	10 per sequence
Histogram binning	All	–	PyRadiomics recommended range (30–130 bins)	54 per sequence
Inter-rater simulation	–	All	Match Inter-rater DICE in [18]	10 per label
Voxel size	All	–	1–1.5 mm, isotropically	10 per sequence
Slice spacing	FLAIR, T2	–	1-4 mm, imaging recommendations in [8]	10 per sequence
	T1, T1c	–	1–1.5 mm, imaging recommendations in [8]	10 per sequence
K-space subsampling	All	–	Random subsampling, factor 0.8–1.0	21 per sequence

Since the radiomic features were extracted on all four MRI sequences and eight tumor labels, the robustness evaluation amounted to more than 16.4 × 10^6^ tests.

Ensuring absolute agreement and not only consistency across perturbations is key for a robust feature set, therefore the Intraclass Correlation Coefficient ICC(2,1) was chosen for robustness evaluation. The cut-off for the lower bound of the 95% confidence interval of the ICC(2,1) was set at 0.85, indicating good reliability according to [28], and following the publication of Lao et al. [13]. We consider a feature robust if it reaches this threshold for all tested perturbations.

### Feature selectors and ML methods

Machine learning models with high-dimensional feature spaces and only a few training samples suffer from the curse of dimensionality [29], which considerably increases the likelihood of poor performance when the ML model is used in practice. We chose thirteen feature selection and twelve ML methods from the literature (see supplementary material, sections S5, S6, and Table S2). The feature selection methods tested include ReliefF (RELF), Fischer Score (FSCR), Gini index (GINI), Chi-square score (CHSQ), joint mutual information (JMI), conditional infomax feature extraction (CIFE), double input symmetric relevance (DISR), mutual information maximization (MIM), conditional mutual information maximization (CMIM), interaction capping (ICAP), t-test score (TSCR, only for binary classification), minimum redundancy maximum relevance (MRMR), and mutual information feature selection (MIFS). The ML methods Nearest Neighbors, Support Vector Classifiers (SVC) with linear and radial basis function (RBF) kernels, Gaussian processes, decision trees, random forests, multilayer perceptrons, AdaBoost, naïve Bayes, quadratic discriminant analysis (QDA), XGBoost, and logistic regression were included. We remark that we tested all combinations of feature selectors and ML models (i.e., 13 × 12 = 156 combinations, 144 for three OS class experiments, since the t-test score is only applicable for binary classification).

The feature selection step was included in the cross-validation to avoid data leakage and overestimating the single-center performance.

Following [13], we further excluded features with zero median absolute deviation (MAD) and a concordance index (C-index) of 0.55 or lower, regarded as non-predictive features. This threshold setting was chosen considering a tradeoff between only retaining the most predictive features and reducing the curse of dimensionality (see supplementary material, Figure S4).

Detailed information regarding the image acquisition parameters, image pre-processing, and perturbations is available in the supplementary material.

### Clinical and data-driven prior knowledge for feature set reduction

We tested two priors to decrease the features set size further: A sequence prior by only using the T1c and FLAIR MRI since these two sequences are predominantly considered by neuroradiologists when assessing pre-operative data. A second prior, referred hereafter as hand-picked, was introduced by limiting the features to the most robust features types as observed during the robustness analysis: Pyradiomics-derived tumor shape, enhancement geometry, centroids, and patient age.

### Statistical analysis

The performance of the ML approaches was measured by the area under the receiver operating characteristics curve (AUC), balanced and unbalanced accuracy, sensitivity, specificity, F1 score, and precision. The best performing model for every OS class boundary was selected based on the AUC. All metrics were recorded during a 10-fold stratified cross-validation [30] on the single-center dataset. All performance metrics are reported as the mean across all splits.

## Results

### Feature robustness

**Fig. 3:**
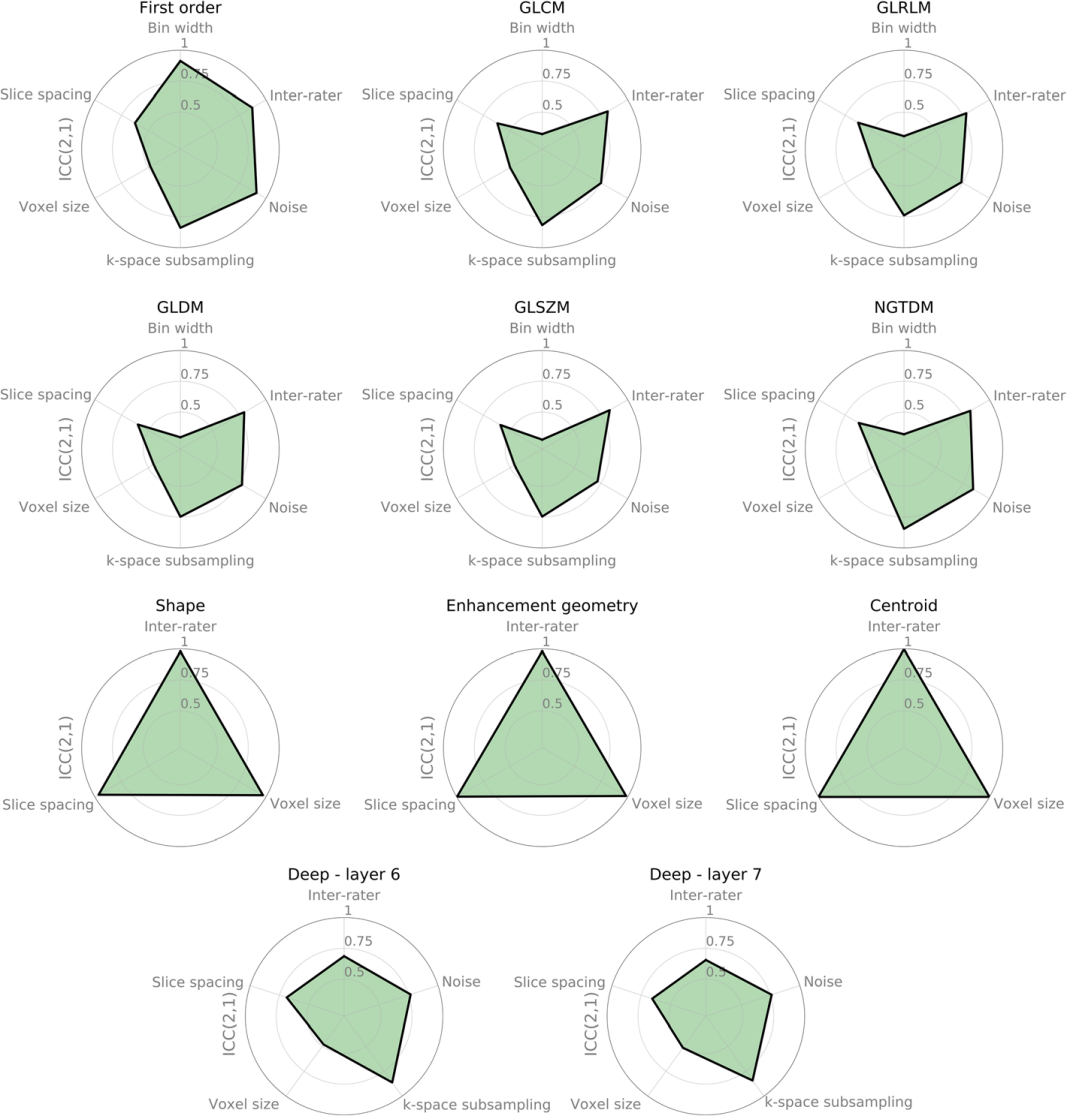
Robustness of radiomic features against perturbations, measured by the intraclass correlation coefficient (ICC(2,1)). The values shown are averages of the lower bound of the 95th percentile of the ICC(2,1) across all segmentation types, MR sequences and features within a given class. Only relevant results are shown, e.g. not showing noise impact for shape features, since no intensity information was used for this feature

Figure 3 shows the mean of the lower bound of 95% confidence level of the ICC(2,1) across MRI sequences and tumor labels per radiomic feature type. The corresponding table with the mean and standard deviation is included in the supplementary material, Table S3. The first-order features from PyRadiomics are most robust against noise and inter-rater variations but have a low agreement if the voxel size or slice thickness is changed. GLCM, GLRLM, GLDM, GLSZM, and NGTDM, describe texture information in the MRI image and show similar behavior. Changes in the binning of gray values used to derive higher-order features have a high negative impact on the agreement, resulting in low robustness levels. Since the gray value binning can be easily controlled and replicated in practice if the whole processing pipeline is well-documented, no feature was excluded based on robustness against gray level bin perturbations. For higher-order features, robustness against noise, k-space subsampling and inter-rater variations of the segmentations, yielded ICC scores around 0.75.

Shape-related feature types (PyRadiomics shape and enhancement geometry) and location features are robust against voxel size, slice spacing changes, and inter-rater variability, with the highest ICC scores across features.

The robustness of features extracted from the two last layers of the pre-trained deep learning model is almost identical (mean ICC values 0.70 and 0.69, and mean standard deviation 0.28 and 0.29 respectively). The ICC was lowest for the voxel size perturbations (ICC = 0.48) and the highest for k-space subsampling (ICC = 0.86). Overall, deep features yielded higher robustness levels than texture-based features, derived from GLCM, GLRLM, GLDM, GLSZM, and NGTDM, but yielded lower ICC values than shape and first-order features.

Only considering the overall robustness of feature classes across labels and MRI sequences is too crude if we want to select individual robust features. Therefore, we considered the robustness of each feature individually. This evaluation for all tested perturbations is available in the supplementary material, Figure S5, and Table S3.

Combining all described feature types on all tumor labels and MRI sequences, a total of 265,604 features were analyzed. With the ICC threshold set to 0.85, 11,306 features (42.5%) remain after robustness testing. The number of robust features for different ICC threshold settings is reported in the supplementary material Figure S6. Features with zero MAD across our training population were removed and considered non-informative, as proposed by [13]. This resulted in a further reduction of features, resulting in 5009 features. An observation we made here was that the deep features were very sparsely populated with non-zero values (47% non-zero for the pre-operative single-center data). If these features are to be used in a machine learning model, only selecting features with a reasonable variability across subjects have to be considered. Based on this observation, we removed all features that are non-zero for only one patient. With this reduction, the set of features further reduced to 3351 features. Since this set still was high-dimensional, we further reduced the number of features by only considering predictive features with a concordance-index higher than 0.55, being less restrictive than [13], since this step was followed by feature selection. With this last constraint, the final set comprises 564 features (0.21% from original feature set), divided into 558 deep, one GLSZM, four shape features, and the age of the patient. Since the number of features was still higher than the number of samples, a feature selection step had to be included before the ML algorithms were added.

### Feature selection and machine learning models

We tested the performance of popular and widely used feature selection methods and ML models on different OS class boundaries similar to [31]. The results for all OS boundaries and classification metrics are included in the supplementary material (Table S4, Figure S7).

The best performing algorithm for each OS class boundary was trained on the whole single-center dataset and applied to the unseen multicenter data (BraTS TCIA).

#### Performance using non-robust features

**Fig. 4:**
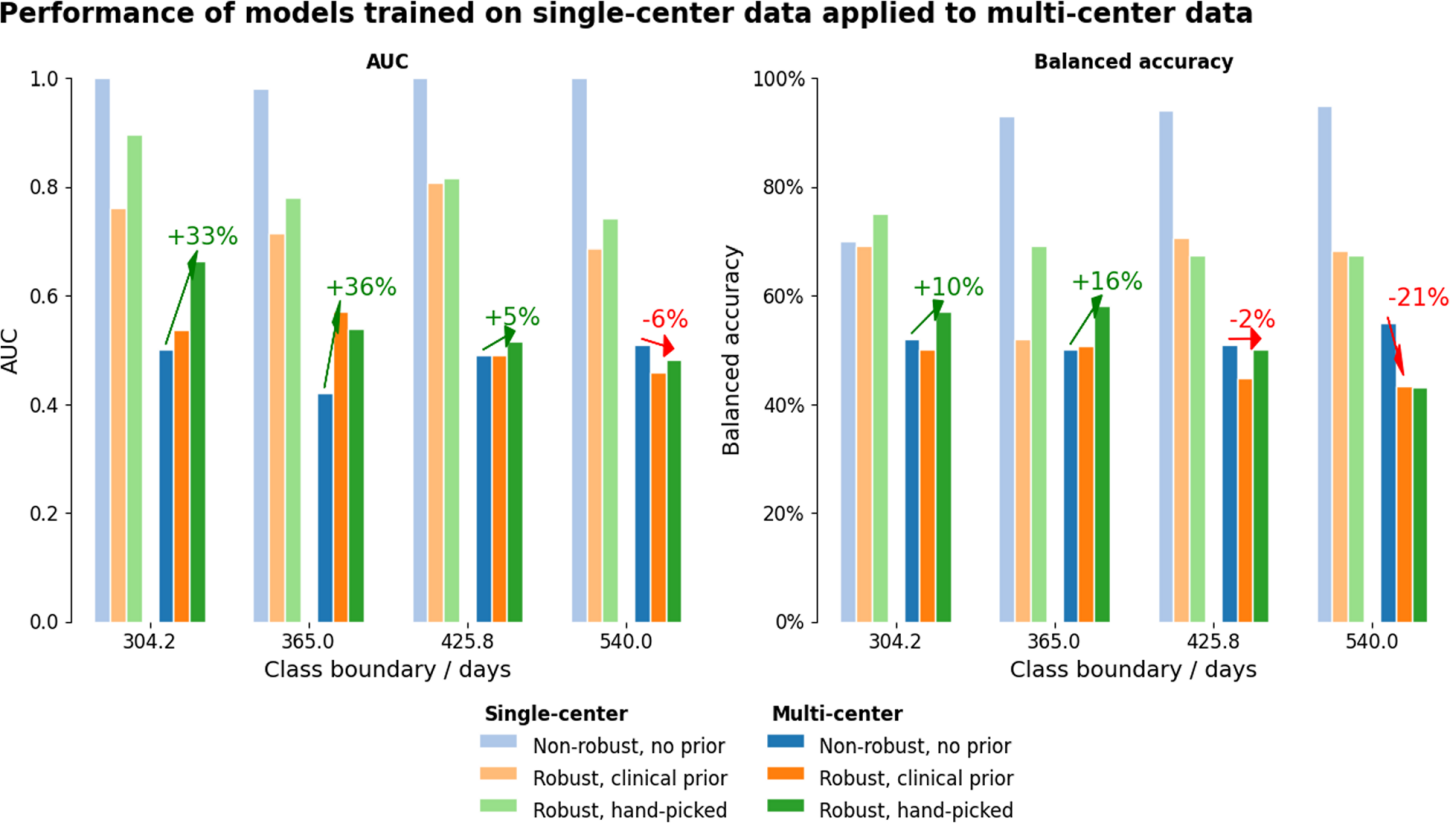
Performance comparison single- versus multi-center for two overall survival classes. Shown for non-robust feature sets, robust features with sequence prior, and hand-picked feature selection. The results show the trade-off between single-center performance and the drop when moving to multi-center data. Introducing priors helped reduce performance drop. The arrows indicate whether a prior increased performance on multi-center data when compared to the non-robust features. The benefit of robust features highly depends on the class boundary used, since different feature selections and machine learning methods were used. The supplementary material contains corresponding plots for further classification performance metrics, as well as the results for the experiments with three overall survival classes

We observed a major decrease in accuracy (average drop of 48%) and AUC (52% average reduction) when applying the single-center models using non-robust features were used for unseen multi-center data. Figure 4, Table 3, and Table 2 summarize the performance with single-center and multi-center data.

**Table 2:** Comparison of best-performing feature selectors (by AUC). Due to the high class imbalance for class boundaries at high and low overall survival values, the balanced accuracy is reported. The single-center metrics are listed as the mean across splits of stratified 10-fold cross-validation. A large performance drop can be observed when the model is tested on unseen multi-center data

Prior	Center	Robustness	Class Boundary	Selector	ML model	AUC	Bal. Acc.
–	S	Non-robust	304.20	MRMR	Gaussian Process	1.00	70%
–	S	Non-robust	365.00	MIFS	MLP	0.98	93%
–	S	Non-robust	425.80	CIFE	Adaboost	1.00	94%
–	S	Non-robust	540.00	MRMR	MLP	1.00	95%
–	M	Non-robust	304.20	MRMR	Gaussian Process	0.50	52%
–	M	Non-robust	365.00	MIFS	MLP	0.42	50%
–	M	Non-robust	425.80	CIFE	AdaBoost	0.49	51%
–	M	Non-robust	540.00	MRMR	MLP	0.51	55%
MR	S	Robust	304.20	RELF	Random Forest	0.76	69%
MR	S	Robust	365.00	RELF	Nearest Neighbors	0.72	52%
MR	S	Robust	425.80	RELF	XGBoost	0.81	71%
MR	S	Robust	540.00	GINI	Decision Tree	0.69	68%
MR	M	Robust	304.20	RELF	Random Forest	0.54	50%
MR	M	Robust	365.00	RELF	Nearest Neighbors	0.57	51%
MR	M	Robust	425.80	RELF	XGBoost	0.49	45%
MR	M	Robust	540.00	GINI	Decision Tree	0.46	43%
H	S	Robust	304.20	CIFE	XGBoost	0.90	75%
H	S	Robust	365.00	MRMR	AdaBoost	0.78	69%
H	S	Robust	425.80	MRMR	AdaBoost	0.82	67%
H	S	Robust	540.00	GINI	AdaBoost	0.74	68%
H	M	Robust	304.20	CIFE	XGBoost	0.66	57%
H	M	Robust	365.00	MRMR	AdaBoost	0.54	58%
H	M	Robust	425.80	MRMR	AdaBoost	0.51	50%
H	M	Robust	540.00	GINI	Adaboost	0.48	43%

Abbreviations: *MR* sequence prior, *H* hand-picked, *S* single-center, *M* multi-center, *Bal. Acc.* balanced accuracy, *MRMR* minimum redundancy maximum relevance, *MIFS* mutual information feature selection, *CIFE* conditional infomax feature extraction, *RELF* ReliefF, *GINI* Gini index, *CMIM* conditional mutual information maximization, *MLP* multi-layer perceptron, *RBF SVC* support vector classifier with radial basis function kernel. The full table with all performance metrics is reported in the supplementary material

The highest AUC achieved was 1.0 for three out of four tested class boundaries (304.2, 425.8, and 540 days) on single-center data, but dropped below 0.51 when the models were applied to the multi-center BraTS data. The top balanced accuracy was 0.95 with an OS boundary of 540 days, which dropped to 0.55 on multi-center data. Sensitivity was at 100% for the 304.2 days OS boundary and dropped slightly to 0.98 on multi-center data. The specificity, on the other hand, varied greatly between OS boundaries, ranging from 0.25 for the OS boundary at 304.2 days and 0.97 for OS classification at 425.8 days.

### Importance of priors for feature set reduction

We observed that overall, trained ML models predominantly selected deep features from the T1 and T2 MR images. This is likely due to the abundance of these feature types during the feature selection process (see supplementary material, Figure S6). However, since these sequences are rarely considered by neuroradiologists assessing GBM pre-operative images, we designed a sequence prior to only select robust features from the T1c and FLAIR MR images. Figure 4 shows the results of this experiment. Overall, such sequence prior enabled an increase in model accuracy on all but the class boundaries with the highest OS (i.e., 540 days). The performance drop on this class boundary may be partly attributed to the higher median survival and different survival time distribution on the single-center compared to the multi-center BraTS dataset (full dataset information in the supplementary material, Table S5).

**Fig. 5:**
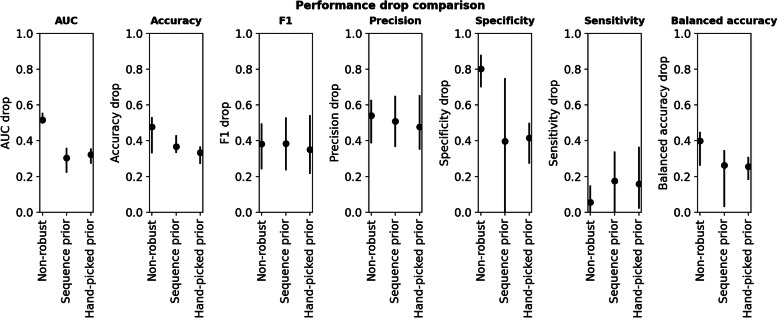
Performance drop comparision across all class boundaries, shown as mean and 95% confidene intervals, calculated with the adjusted bootstrap percentile (BCa) method. Lower is better. The drop for the models using robust features is lower for all recorded metrics, except for the sensitivity. Values are reported in Table 3

We tested a second prior only using the robust feature classes alongside the patient age. Tables 2 and 3 compare the performance of robust features with these priors with the performance obtained when using the full feature set with non-robust features. Using the sequence prior, the average AUC could be improved by 21.1% compared to using the non-robust feature on the unseen multi-center data. The use of the hand-picked (data-driven) prior also improved the balanced accuracy by 14.5% when compared to using the full feature set. While the specificity drop can be greatly improved by using both priors (sequence prior: 40.37%, hand-picked feature prior: 38.35%), the sensitivity decreased for the two OS classification experiments (sequence prior: − 12.19%, hand-picked prior: − 9.81%). The confidence intervals for all metrics can be found in Table 3 and Fig. 5.

**Table 3:** Performance drop of models trained on single-center data and applied to unseen multi-center data, using non-robust and robust featues withs priors, averaged across class boundaries (lower is better). Listed as mean and 95% confidence intervals, calculated with the adjusted bootstrap percentile (BCa) method. The lowest drop is indicated in bold for each metric. Bal. Acc.: Balanced accuracy, Acc.: Accuracy

Feature set	AUC drop	Bal. acc. drop	Acc. drop	Specificity drop	Sensitivity drop	F1 drop	Precision drop
Non-robust features	0.52 CI: [0.50,0.56]	0.40 CI: [0.26,0.45]	0.48 CI: [0.33,0.53]	0.80 CI: [0.70,0.88]	**0.06** CI: [0.00,0.15]	0.38 CI: [0.24,0.50]	0.54 CI: [0.39,0.63]
Robust features, sequence prior	**0.30** CI: [0.22,0.36]	**0.26** CI: [0.03,0.35]	0.37 CI: [0.33,0.43]	**0.40** CI: [−0.10,0.75]	0.18 CI: [0.00,0.34]	0.38 CI: [0.24,0.53]	0.51 CI: [0.37,0.65]
Robust features, hand-picked	0.32 CI: [0.27,0.36]	**0.26** CI: [0.18,0.31]	**0.33** CI: [0.27,0.37]	0.42 CI: [0.27,0.50]	0.16 CI: [0.02,0.37]	**0.35** CI: [0.22,0.54]	**0.48** CI: [0.35,0.66]

## Discussion

Previous studies demonstrated how selected perturbations affect radiomic features, e.g., [32–35], or assessed the performance of feature selection techniques together with a wide range of ML methods (e.g., [31]). In this study, we combine multiple image degradation methods in order to robustify a radiomic signature for the application of GBM survival classification. Since previous studies tested machine learning methods for this task on many different OS class boundaries, we conducted a high-throughput analysis with over 16.4 × 10^6^ tests on feature robustness and evaluated 156 combinations of popular feature selectors and machine learning techniques for seven overall survival class boundaries. Additionally, we tested the value of introducing priors based on a) clinical practice and b) hand-picking robust non-intensity features based on literature reports and own observations.

The results show that very high AUC values can be achieved in the single-center setting for the non-robust feature set. Transferring the model to the multi-center BraTS data caused a large drop in all assessed performance metrics. Introducing a sequence prior to using only the T1c and FLAIR images resulted in a lower drop when moving to multi-center data. The benefit of introducing this prior indicates that the feature selection techniques may suffer from the multiple-testing issue. To further reduce the number of features entering the selection process, we performed another test only using the most robust hand-picked non-intensity features alongside age (PyRadiomics shape features, enhancement geometry, and tumor centroids). The models trained with this more restrictive hand-picked feature prior outperformed the sequence prior for AUC in a single-center setting and resulted in a higher or equal multi-center performance for all class boundaries for AUC, balanced accuracy, and F1-score. Higher performance than that obtained using the sequence prior could be achieved for accuracy (for three out of four class boundaries), sensitivity (for two out of four class boundaries), and specificity (three out of four class boundaries), further highlighting the importance of incorporating domain knowledge for the design of robust and meaningful features, as opposed to utilizing a pure data-driven feature extraction approach.

The sensitivity drop for longer OS obtained when using robust features may be partly explained by the different OS distributions on the single-center training data (OS: 22.77 ± 14.74 months) and the BraTS data (OS: 14.78 ± 11.98 months). Therefore, the requirements regarding sensitivity or specificity for a given application have to be carefully evaluated.

### Limitations

The perturbations selected for this study are motivated by the imaging recommendations by [8] and were not selected based on the target dataset. Since some of the BraTS data includes cases acquired ten or more years prior, image quality is lower than recently acquired MRI with the latest technology. This leads to better applicability for further research with more recently acquired MRI, but a lack of specificity regarding the expected image quality on the BraTS data.

Not all perturbations could be selected based on imaging recommendations but had to be hand-tuned and visually inspected. Similarly, the applied k-space subsampling approximates real subsampling during acquisition since the phase information is already lost.

Furthermore, the sample size was rather small for both single- and multi-center data, with different overall survival distributions.

## Conclusions

These results demonstrate that if a model is to be robustified before deploying it on unseen multi-center data, a trade-off between single-center accuracy and reduction of performance drop is possible, but as shown here, it can lead to a performance benefit when applied to multi-center data, as compared to using a non-robust set that might seem optimal during the development phase. Furthermore, we show that inclusion of prior knowledge, through the selection of MRI sequences or which type of robust features are used, helps to reduce the number of considered features, and partly alleviates the multiple-testing problem. The remaining performance drop calls for adhering to imaging guidelines and great care when transferring ML models to unseen data. With the rise of new deep learning techniques such as style transfer and improved normalization (e.g., [36]), this issue could hopefully be further alleviated.

We derive the following recommendations for future work on GBM patient survival classification:

- The choice of OS class boundaries should be clearly motivated, e.g., data-driven or to ensure comparability to previous studies. We encourage testing and reporting newly proposed techniques on multiple class boundaries.
- The perturbations considered for feature robustness testing should be tailored to the target dataset and in line with imaging guidelines. If the imaging parameters used for the target dataset are known, the perturbation types and magnitudes should be tuned accordingly.
- A broader set of classification performance metrics should be reported apart from AUC and accuracy, and along with a description of data distribution. Not providing a thorough report of metrics and data distribution can be misleading for other researchers evaluating the advantages and drawbacks of a given proposed method.
- With convenient libraries for feature extraction, selection, and machine learning, great care should still be taken regarding, e.g., multiple-testing problems, and introducing prior domain knowledge is still of high value.

We believe the proposed tool of radiomic feature robustness testing is applicable to other modalities, outcomes, and diseases, with certain perturbations being modality-specific. For CT-based radiomics, we expect the robustness to profit from the quantitative nature of the imaging data, in comparison to MRI-based radiomics. Nonetheless, considerable variability for CT radiomics has been reported recently for inter-vendor, and intra-subject test-retest studies [37]. For CT-based radiomics, e.g., the k-space subsampling perturbation type can be exchanged with a kernel-based reconstruction perturbation. Adapting to other diseases may include switching to other modalities and appropriate outcome metrics. We recommend a careful evaluation as to how the physiology of the disease affects radiological findings from imaging data.

## Supplementary information

**Additional file 1.** The supplementary material contains further information on the data used, methods, software tools and versions, and additional experiment results to improve reproducibility.

## Data Availability

The multi-center dataset used in this study is publicly available through the Multimodal Brain Tumor Segmentation Challenge (BraTS) on http://braintumorsegmentation.org/. We are not allowed to share the single-center data used. The code used for the robustness evaluation and machine learning method comparison is available at https://github.com/ysuter/gbm-robustradiomics, details on pre-processing and method parameter tuning ranges are listed in the supplementary material.

## Abbreviations

AUC: Area under the curve, here: the receiver operating curve (ROC)
BraTS: Brain tumor segmentation challenge
C-Index: Concordance index
cet: contrast-enhacement (tumor segmentation label)
CHSQ: Chi-square (feature selection)
CIFE: Conditional infomax feature extraction (feature selection)
CMIM: Conditional mutual information maximisation (feature selection)
CNN: Convolutional Neural Network
DISR: Double input symmetric relevance (feature selection)
DL: Deep learning
ed: Edema (tumor segmentation label)
FLAIR: Fluid attenuated inversion recovery (MRI)
FSCR: Fisher score (feature selection)
GBM: Glioblastoma multiforme
GINI: Gini index (feature selection)
ICAP: Interation capping (feature selection)
ICC: Intraclass correlation coefficient
JMI: Joint mutual information (feature selection)
MAD: Median absolute deviation
MIFS: Mutual information feature selection
MIM: Mutual information maximization (feature selection)
ML: Machine learning
MLP: Multilayer Perceptron
MRMR: Minimum redundancy maximum relevance
nec: Necrosis (tumor segmentation label)
net: Non-enhancing tumor (tumor segmentation label)
net_ed: Non-enhancement combined with the edema (tumor segmentation labels)
net_ncr: Necrosis and non-enhancement combined (tumor segmentation labels)
OS: Overall survival
QDA: Quadratic discriminant analysis
RBF: Radial basis function
RELF: RelieF (feature selection)
ROC: Receiver operating characteristic
SNR: Signal-to-noise ratio
SVC: Support vector classifier
T1: T1-weighted (MRI)
T1c: T1-weighted with contrast agent (MRI)
T2: T2-weighted
TCIA: The cancer imaging archive
TSCR: T-score (feature selection)
wt: Whole tumor (tumor segmentation label)
