# Supplementary material for: Radiomics for glioblastoma survival analysis in pre-operative MRI: exploring feature robustness, class boundaries, and machine learning techniques
Source: Cancer Imaging. 2020 Aug 5;20:55. doi: 10.1186/s40644-020-00329-8 (PMC7405445; doi:10.1186/s40644-020-00329-8)
Supplement: Supplementary file 1 — Additional file 1. The supplementary material contains further information on the data used, methods, software tools and versions, and additional experiment results to improve reproducibility. [file 40644_2020_329_MOESM1_ESM.docx]

**­­Supplementary Material for:**

# Radiomics for Glioblastoma Survival Analysis in Pre-operative MRI: Exploring Feature Robustness, Class Boundaries, and Machine Learning Techniques

We provide additional information regarding data used, its preparation and processing, as well as details on the evaluation.

## S1 Data

Figure S1 compares the distributions of the overall survival time and the patient age for the single- and multi-center data used. The acquisition parameters per sequence are listed in Table S5.


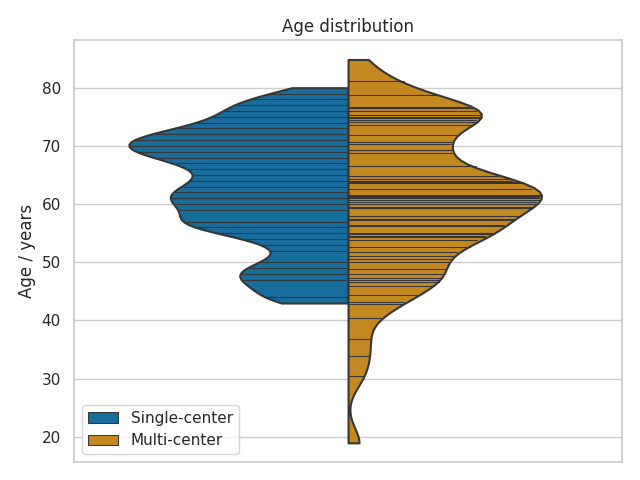

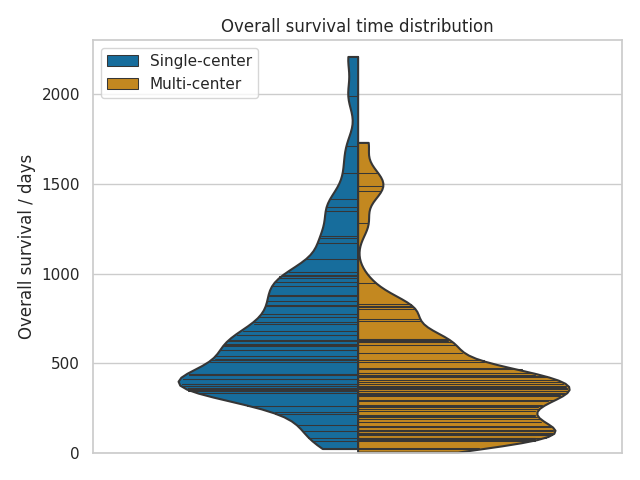


**Figure S1**: Age and overall survival time distribution comparison for single-center (blue) and multi-center (brown) data used. Left: Age distribution: Single-center data with a narrower age range compared to the multi-center BraTS TCIA data. Right: Overall survival distributions of the single- and multi-center data. The single-center data contains more long survivors, while the peak of the most frequently occurring survival times are close at approximately 400 days.

## S2 Software

All image processing and machine learning experiments were performed using Python 3.6. Relevant libraries include Pymia 0.2.1, PyRadiomics 2.2.0^1^, SimpleITK 1.2.0^2^, Rpy2 3.0.5, Scikit-feature 1.0.0^3^, and Scikit-learn 0.21.3^4^.

Skull-stripping and tumor segmentation was performed with BraTuMIA version 2.0.0.8^5,6^.

## S3 Pre-Processing

### Single-center data

After retrieving the anonymized MRI in the DICOM format, all images were converted to the Nifty-format. Resampling to 1mm isovoxels, skull-stripping, registration to the T1c image, and tumor segmentation was performed with BraTuMIA. Bias field correction was performed using the N4BiasFieldCorrectionImageFilter^7^ from SimpleITK.

### Multi-center data

The multi-center BraTS data was retrieved as provided by the challenge organizers for the 2018 edition. Bias field correction and tumor segmentation were performed as described for the single-center data.

Affine registration to MNI305 atlas space was performed using *mri_robust_register*^8^, using moments to initialize the orientation and cubic B-splines for interpolation.

## S4 Perturbations

Voxel size and axial slice spacing:
Feature extraction with different voxel sizes and slice spacings was set through the provided settings file for the PyRadiomics feature set. The PyRadiomics pipeline was altered to save the resampled images for further use for the deep, centroid, and enhancement geometry features.

### K-space subsampling

The original image was transformed to the frequency domain using the ForwardFFT filter in SimpleITK. The resulting image was masked using a randomly generated mask to set a fixed amount of frequencies to zero. The masked image was transformed back using the InverseFFT filter. The minimum subsampling rate was determined by visual inspection, approximately matching lower expected image quality encountered on the multi-center data.

### Inter-rater segmentation variability

Due to the lack of multi-rater data for the given tumor segmentation task, this perturbation was artificially created as well. The output segmentation from BraTuMIA was slightly deformed using B-Spline transforms with the control points per image dimension, nearest-neighbor interpolation, spatial rank two and zero fill value.

Since we use eight tumor labels, published inter-rater DICE scores were not available for each. We, therefore, used combined values, as shown in Table S1. For matching the deformed labels to the desired inter-rater DICE and standard deviation, the DICE value was given twice the weight of the standard deviation.

**Table S1:** Inter-rater DICE values and reported standard deviation between human raters. The elastic deformation outputs were evaluated to closely match these values to simulate inter-rater variability.

| **Label** | **DICE** | **DICE standard deviation** |
| --- | --- | --- |
| Contrast-enhncement (cet) | 0.74 | 0.13 |
| Tumor core (core) | 0.93 | 0.03 |
| Whole tumor (wt) | 0.88 | 0.02 |
| Non-enhancing tumor (net) | 0.93 | 0.03 |
| Edem (ed) | 0.88 | 0.02 |
| Necrosis (nec) | 0.93 | 0.03 |
| Non-enhacing tumor and necrosis combined (net_ncr) | 0.93 | 0.03 |
| Non-enhacing tumor and edema combined (net_ed) | 0.905 | 0.025 |


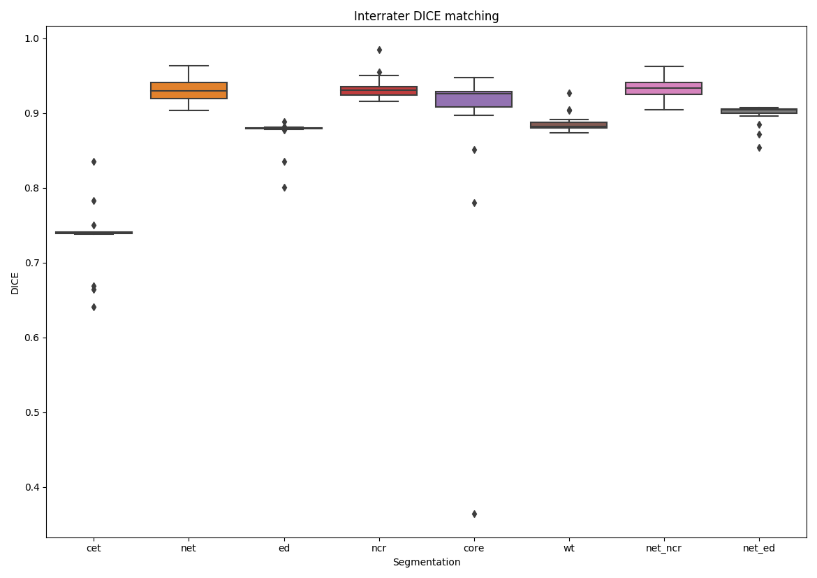


**Figure S2** DICE scores for the artificially created segmentation variations, matching the values for the mean and standard deviation from Table S1.

### Noise

The signal-to-noise ratio (SNR) was measured per MR sequence on the subset of 19 patients used for robustness testing. The SNR was calculated from the mean intensity of the white matter (segmentation obtained through BraTuMIA) and the standard deviation of the background intensity. The background mask was derived from the non-skull-stripped image through thresholding and subsequent hole-filling (radius=20). With the Rayleigh correction, the SNR was calculated as

$SNR=\sigma_{R} \frac{\bar{I}_{WM}}{\sigma_{BG}}$,

where $\sigma_{R}$ is the Rayleigh correction factor (0.655), $\bar{I}_{WM}$the mean white matter intensity, and $\sigma_{BG}$the standard deviation of the background. For the noise perturbation, additive Gaussian noise was added, such that the SNR did not exceed the recorded SNR by more than one standard deviation above the measured mean.

Figure S3 shows the SNR distribution for all four MR sequences on the 19 patient subset used for robustness testing.


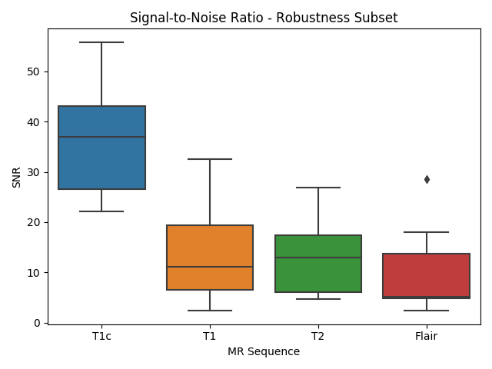


**Figure S3**: Signal-to-noise ratio for the individual MR sequences, evaluated on the single-center data subset considered for the robustness testing (19 patients).

## 5

4

**4**

|  | |
| --- | --- |
|  |  |
|  |  |
|  |  |
|  |  |
|  |  |
|  |  |
|  |  |
|  |  |
|  |  |
|  |  |
|  |  |
|  |  |
|  |  |
|  |  |

### 6

## S7 Robustness Testing

Feature robustness was evaluated using the lower bound of the 95^th^ percentile range of the ICC(2,1). The ICC was calculated using the “psych” package in R 3.6.1 through rpy2.

Figure S6 shows the number of robust features for different ICC thresholds. We observed that deep features from the T1 and T2 MR images were selected predominantly. This is likely due to the abundance of these feature types compared to the PyRadiomics and enhancement geometry features.

Figure S5 shows the variability of feature robustness within a class for the tested perturbations.

**Figure S5** Variability of feature robustness within feature classes for the tested perturbations. Robustenss is measured by the lower bound of the 95^th^ percentile of the ICC(2,1). Considering only mean values across tumor labels is not enough, each feature has to be assessed individually for every label. Across all feature types, the features calculated on the necrotic, and the combination of necrotic and non-enhancing tumor areas are the most robust. Cet: contrast-, area, core: tumor core, ncr: necrosis, net: non-enhancing tumor, ed: edema, wt: whole tumor (including edema), net_ncr: non-enhancement and necrosis combined, net_ed: non-enhacement and edema combined.


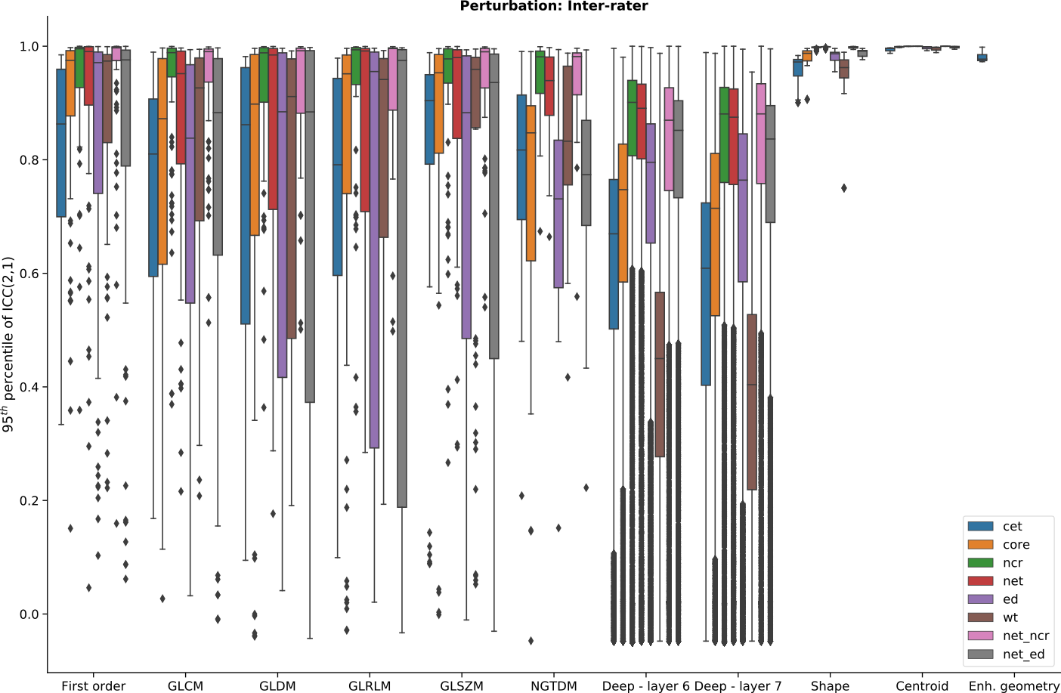

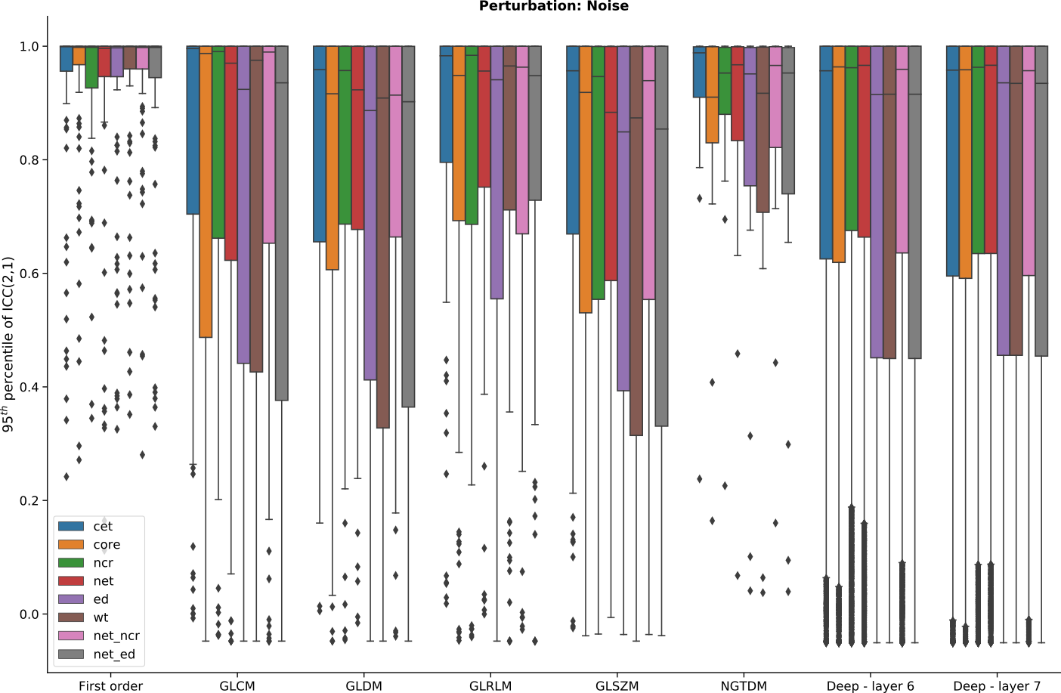

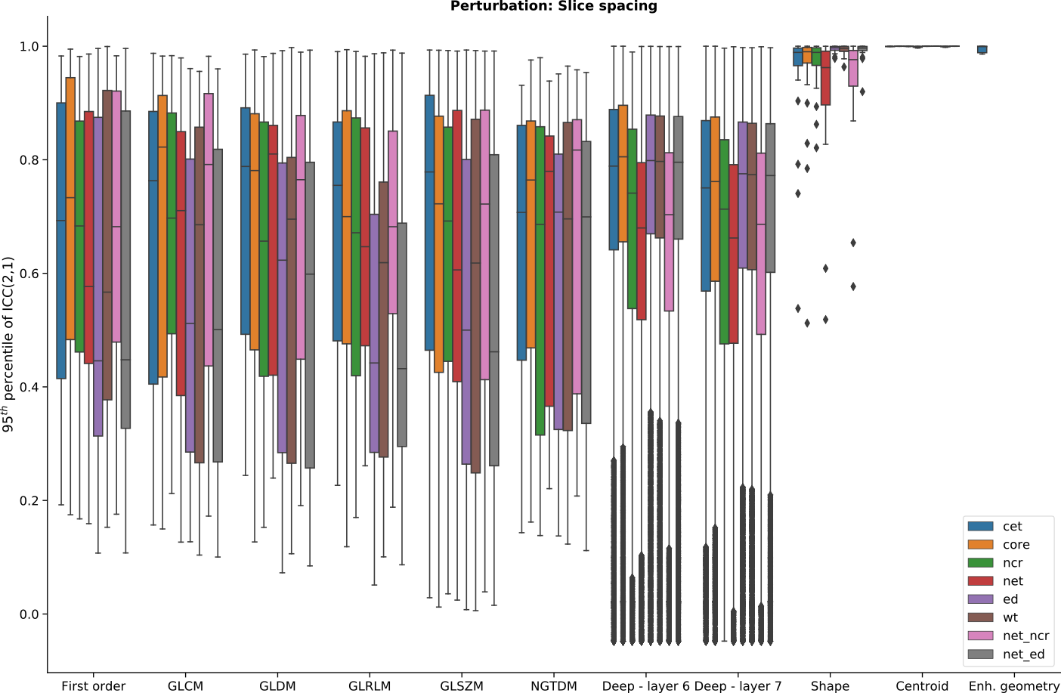

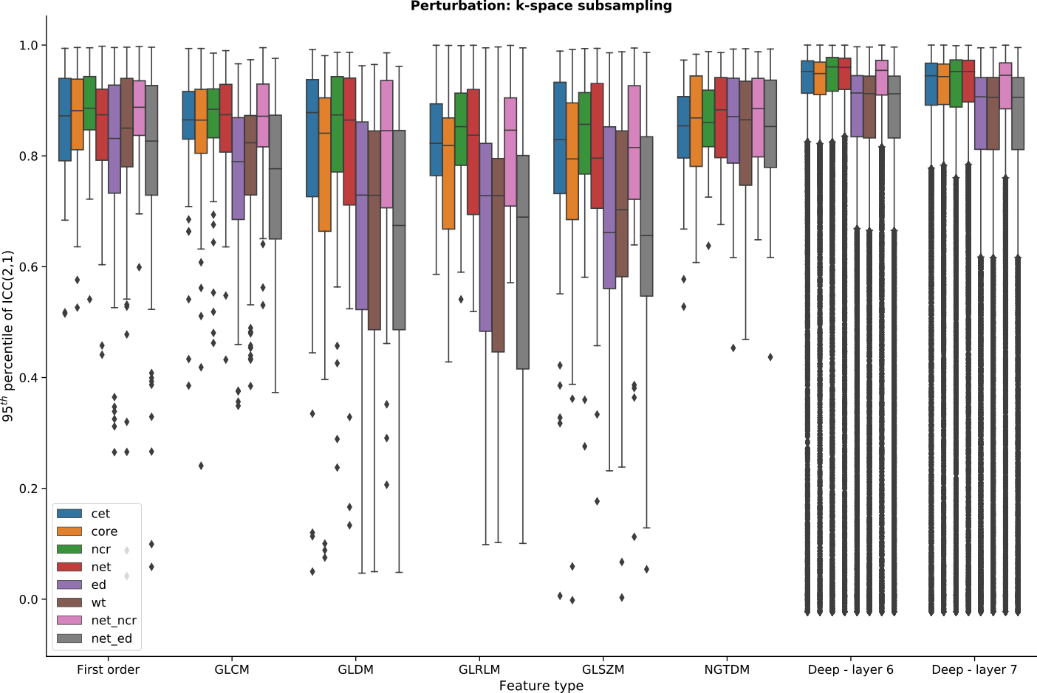

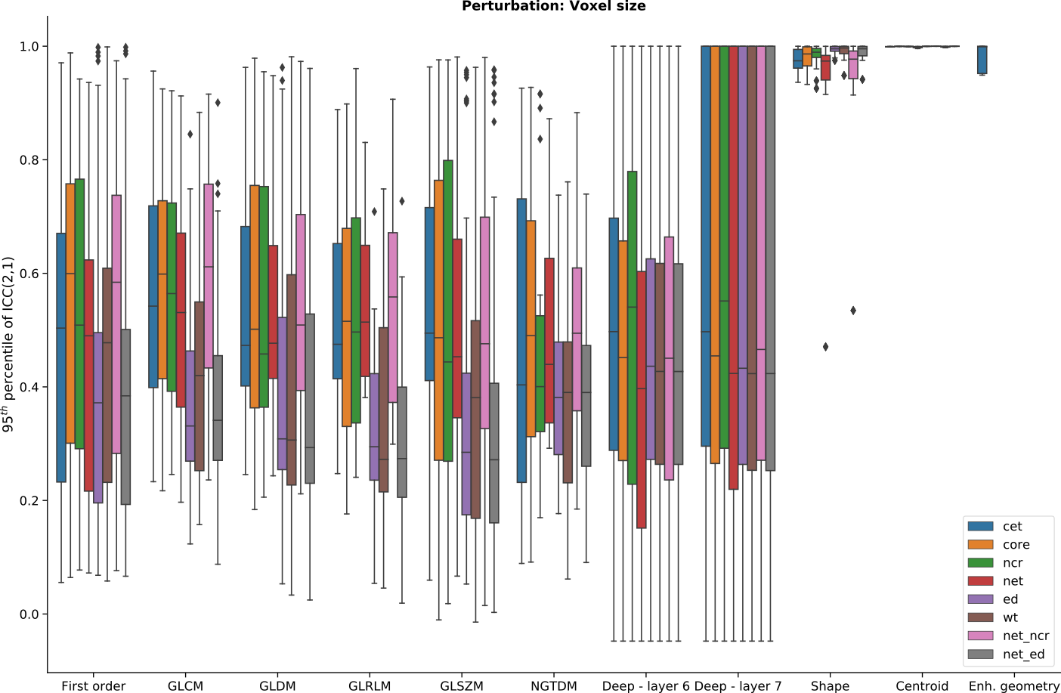


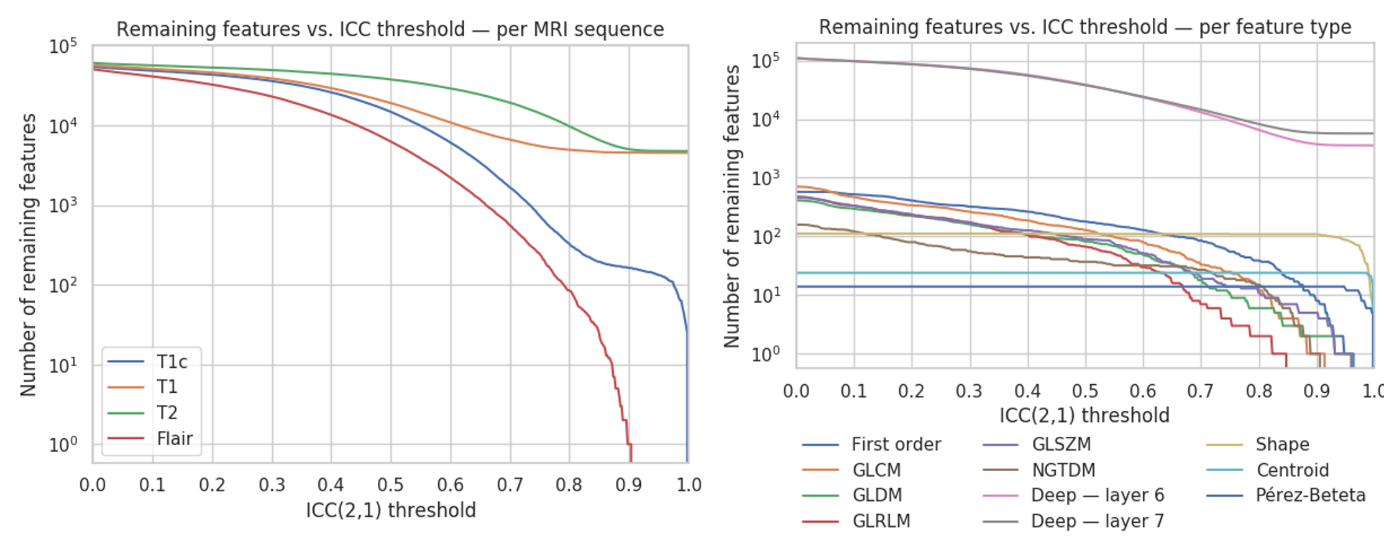


**Figure S6** Number of remaining features with changing the ICC threshold. Left: Remaining features per MRI sequence, showing high robustness for features derived from T1 and T2 images. Right: Remaining features per feature type, showing the predominance of deep features.

|  | |
| --- | --- |
|  |  |
|  |  |
|  |  |
|  |  |
|  |  |
|  |  |
|  |  |
|  |  |
|  |  |
|  |  |
|  |  |
|  |  |
|  |  |
|  |  |

**Table S3**: Lower bound of the 95^th^ percentile of the ICC(2,1) for each feature class and perturbation, listed as mean **±** standard deviation across all features of a given class.

| **Type** | **Perturbation** | **ICC(2,1) 95^th^ Percentile, lower bound**  mean ± standard deviation |
| --- | --- | --- |
| Enhacement geometry | Inter-rater | 0.98 ± 0.01 |
| Enhacement geometry | Voxel size | 0.98 ± 0.02 |
| Enhacement geometry | Slice spacing | 1.00 ± 0.01 |
| Centroid | Inter-rater | 1.00 ± 0.00 |
| Centroid | Voxel size | 1.00 ± 0.00 |
| Centroid | Slice spacing | 1.00 ± 0.00 |
| First order | Bin width | 0.91 ± 0.25 |
| First order | Noise | 0.91 ± 0.18 |
| First order | Inter-rater | 0.87 ± 0.21 |
| First order | k-space subsampling | 0.84 ± 0.15 |
| First order | Voxel size | 0.48 ± 0.26 |
| First order | Slice spacing | 0.62 ± 0.28 |
| GLCM | Bin width | 0.32 ± 0.34 |
| GLCM | Noise | 0.75 ± 0.36 |
| GLCM | Inter-rater | 0.81 ± 0.24 |
| GLCM | k-space subsampling | 0.82 ± 0.14 |
| GLCM | Voxel size | 0.50 ± 0.20 |
| GLCM | Slice spacing | 0.62 ± 0.27 |
| GLDM | Bin width | 0.31 ± 0.24 |
| GLDM | Noise | 0.74 ± 0.35 |
| GLDM | Inter-rater | 0.78 ± 0.30 |
| GLDM | k-space subsampling | 0.74 ± 0.23 |
| GLDM | Voxel size | 0,49 ± 0,23 |
| GLDM | Slice spacing | 0,63 ± 0,27 |
| GLRLM | Bin width | 0,30 ± 0,26 |
| GLRLM | Noise | 0,78 ± 0,34 |
| GLRLM | Inter-rater | 0,80 ± 0,30 |
| GLRLM | k-space subsampling | 0,75 ± 0,20 |
| GLRLM | Voxel size | 0,45 ± 0,20 |
| GLRLM | Slice spacing | 0,60 ± 0,26 |
| GLSZM | Bin width | 0,28 ± 0,23 |
| GLSZM | Noise | 0,72 ± 0,35 |
| GLSZM | Inter-rater | 0,83 ± 0,26 |
| GLSZM | k-space subsampling | 0,75 ± 0,20 |
| GLSZM | Voxel size | 0.46 ± 0.27 |
| GLSZM | Slice spacing | 0.59 ± 0.31 |
| Deep layer 6 | Noise | 0.77 ± 0.31 |
| Deep layer 6 | Inter-rater | 0.69 ± 0.27 |
| Deep layer 6 | k-space subsampling | 0.86 ± 0.22 |
| Deep layer 6 | Voxel size | 0.48 ± 0.33 |
| Deep layer 6 | Slice spacing | 0.69 ± 0.25 |
| Deep layer 7 | Noise | 0.76 ± 0.32 |
| Deep layer 7 | Inter-rater | 0.66 ± 0.29 |
| Deep layer 7 | k-space subsampling | 0.84 ± 0.23 |
| Deep layer 7 | Voxel size | 0.52 ± 0.35 |
| Deep layer 7 | Slice spacing | 0.66 ± 0.26 |
| NGTDM | Bin width | 0.32 ± 0.34 |
| NGTDM | Noise | 0.85 ± 0.24 |
| NGTDM | Inter-rater | 0.82 ± 0.20 |
| NGTDM | k-space subsampling | 0.85 ± 0.12 |
| NGTDM | Voxel size | 0.46 ± 0.22 |
| NGTDM | Slice spacing | 0.62 ± 0.27 |
| Shape | Inter-rater | 0.98 ± 0.03 |
| Shape | Voxel size | 0.97 ± 0.07 |
| Shape | Slice spacing | 0.98 ± 0.05 |

## S8 Single-Center Performance evaluation

Only the top performing method is reported in the main document. In the following sections, we provide the results for all class boundaries as the mean of the 10-fold cross-validation on the single-center data.

Please note that in the few cases where the metric field is empty for the QDA classifier, no posterior probabilities were returned by the estimator.

### Two Overall Survival Classes


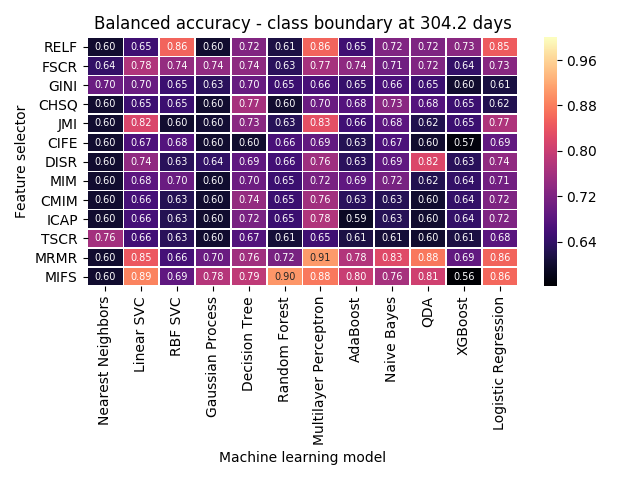

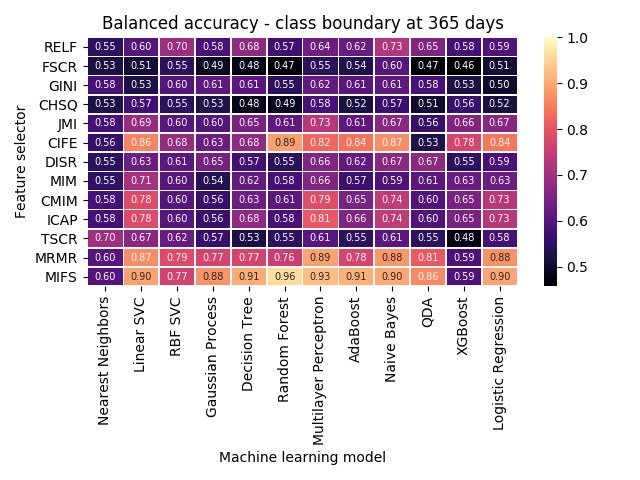

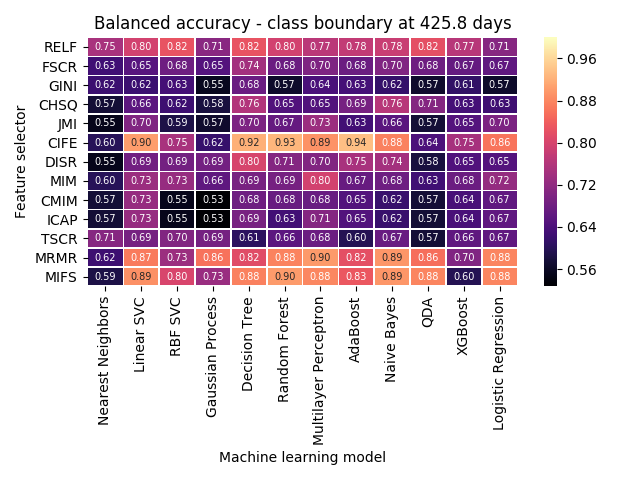

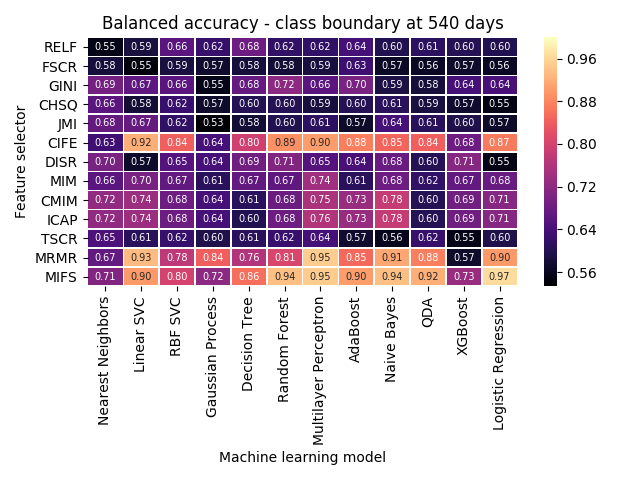

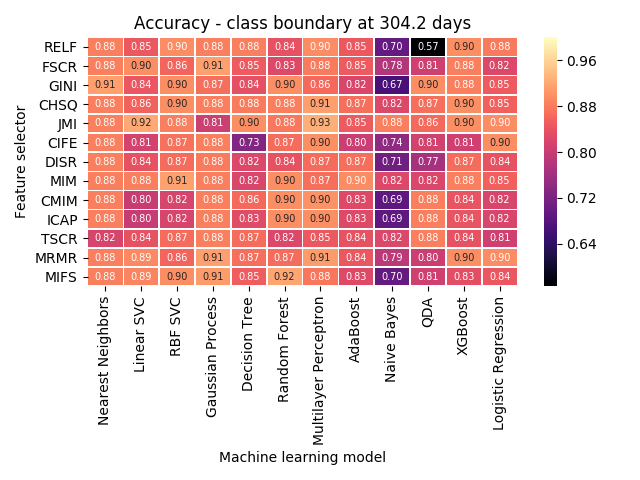

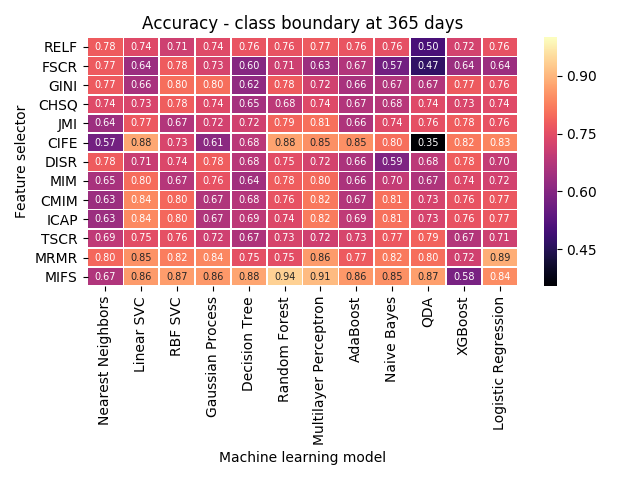

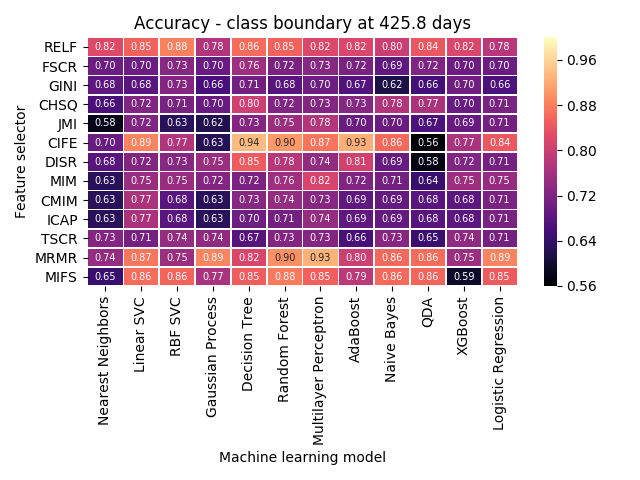

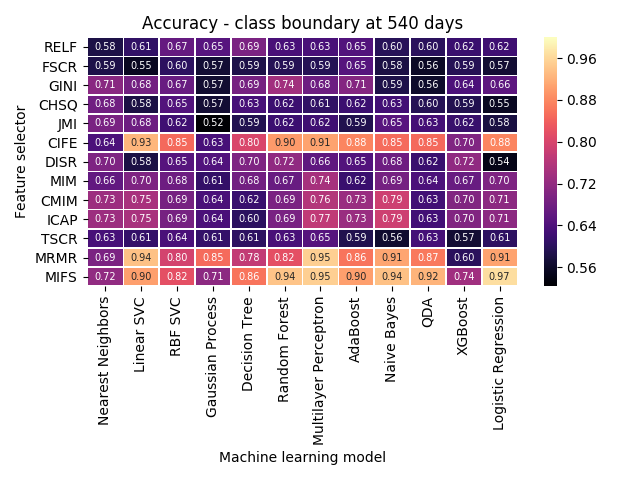

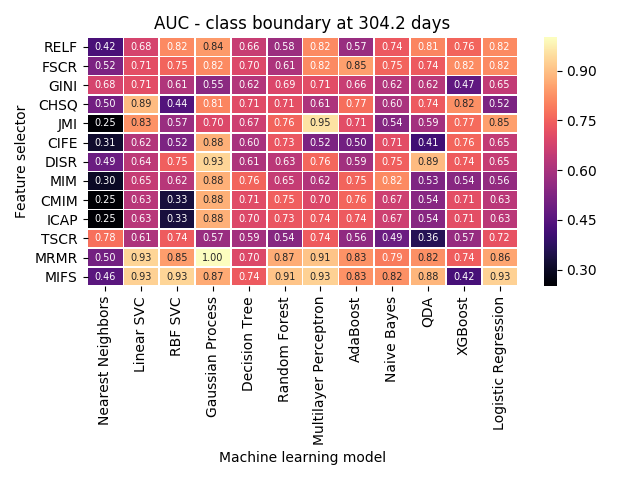

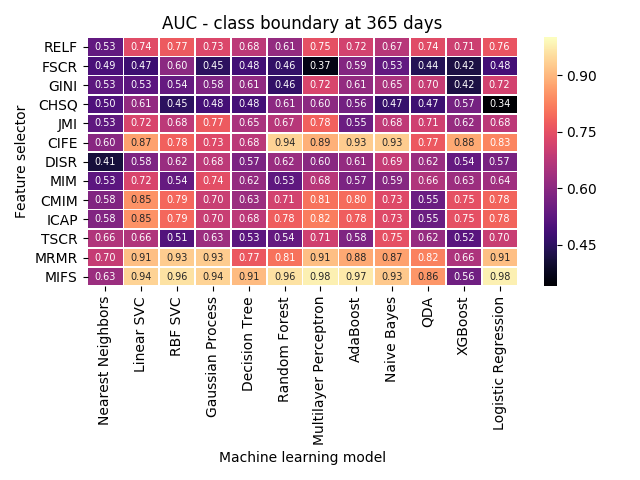

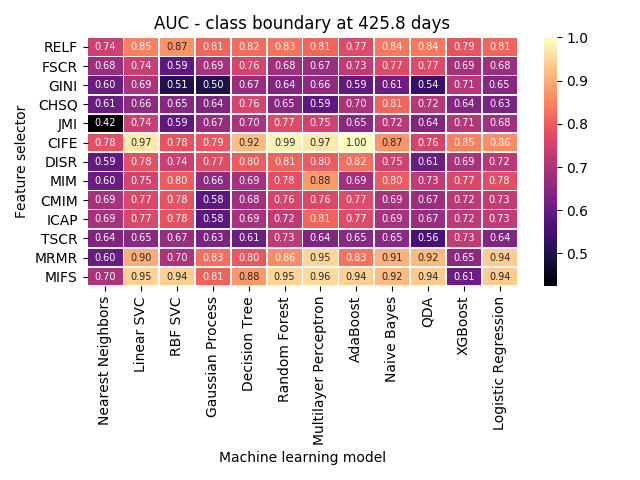

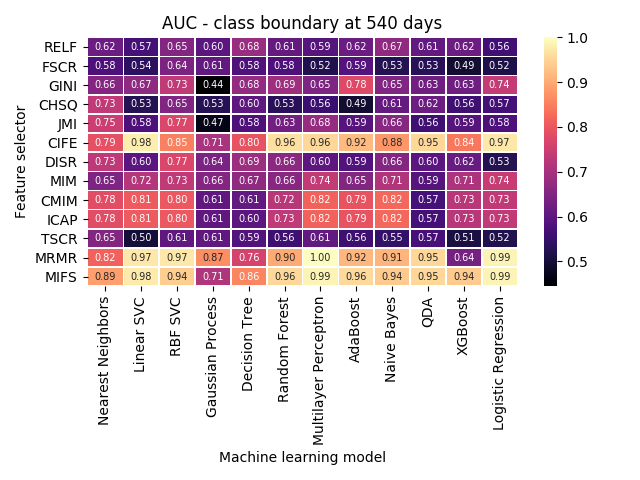

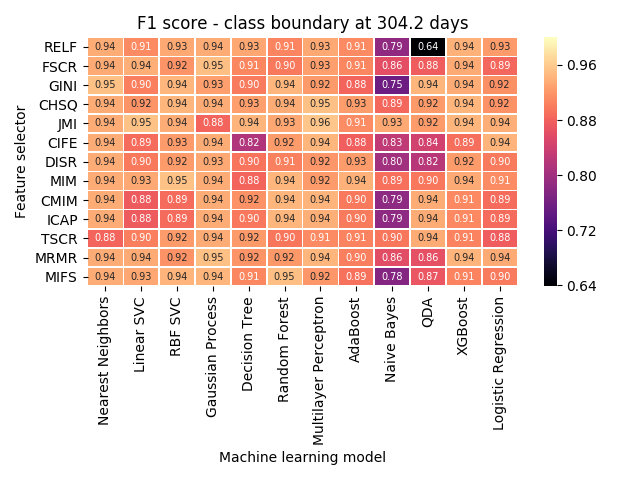

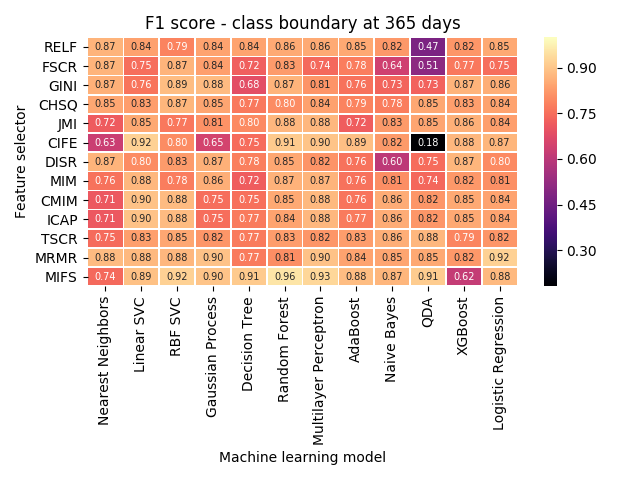

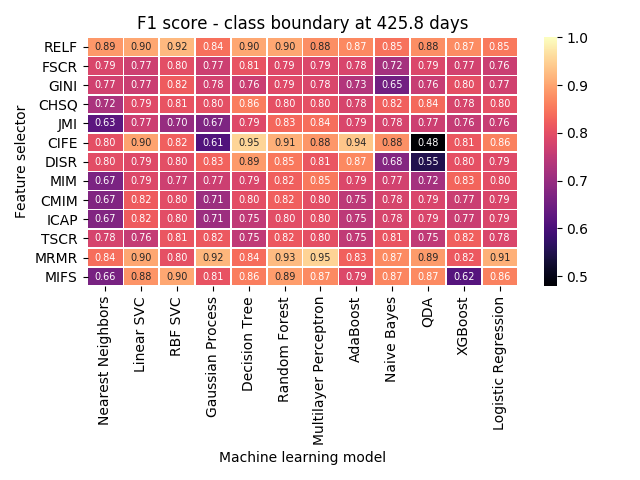

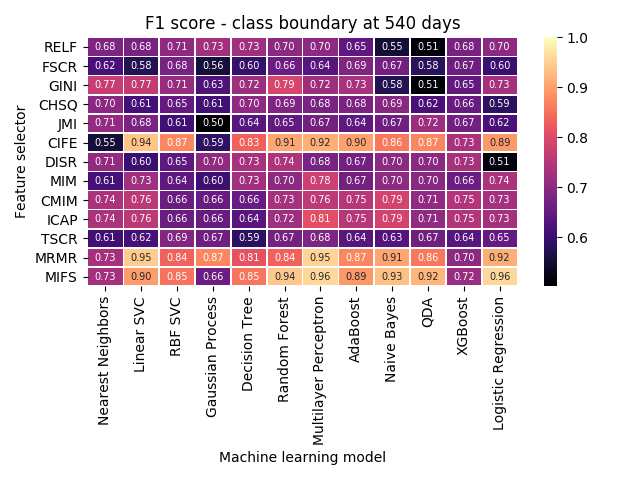

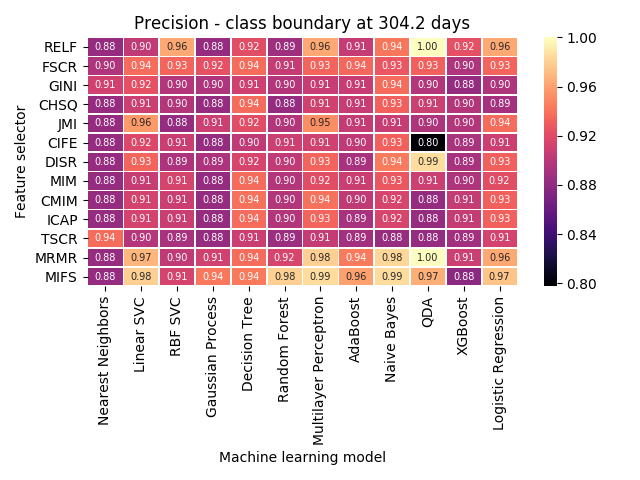

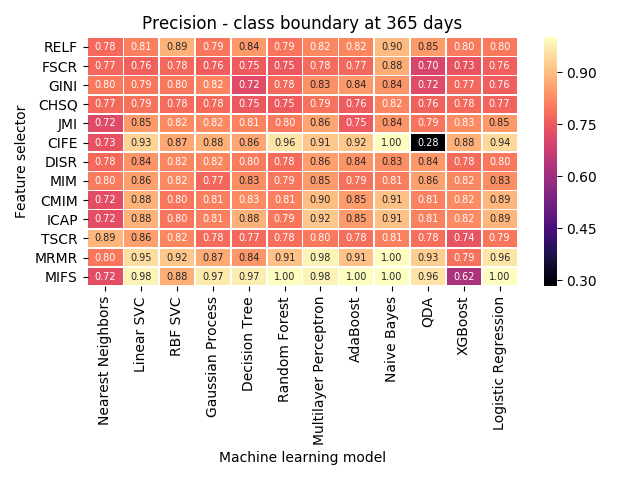

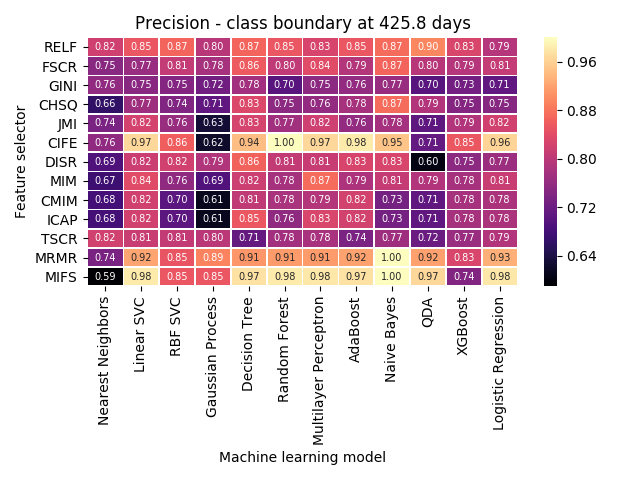

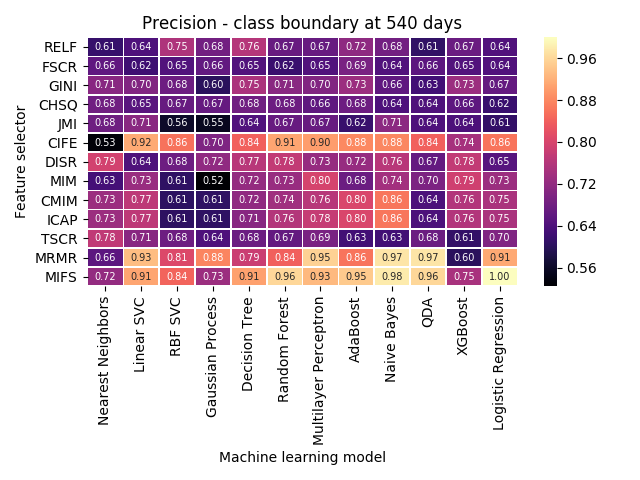

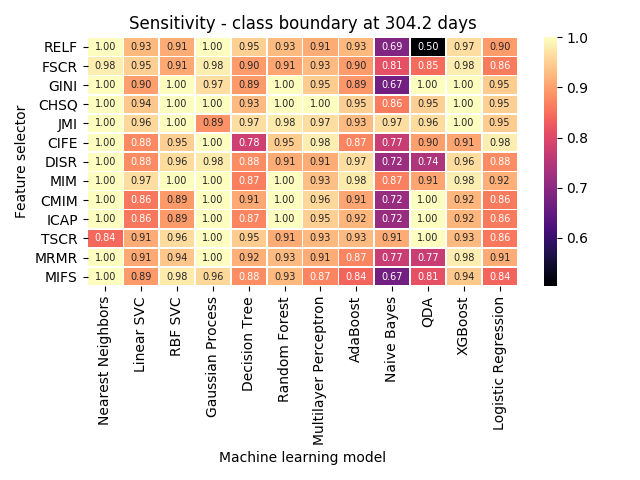

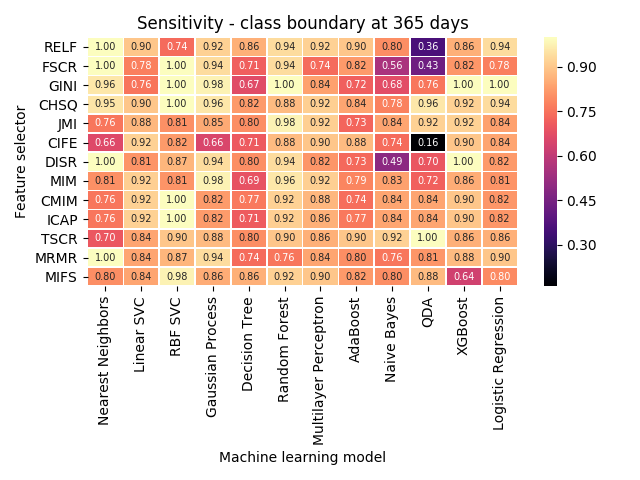

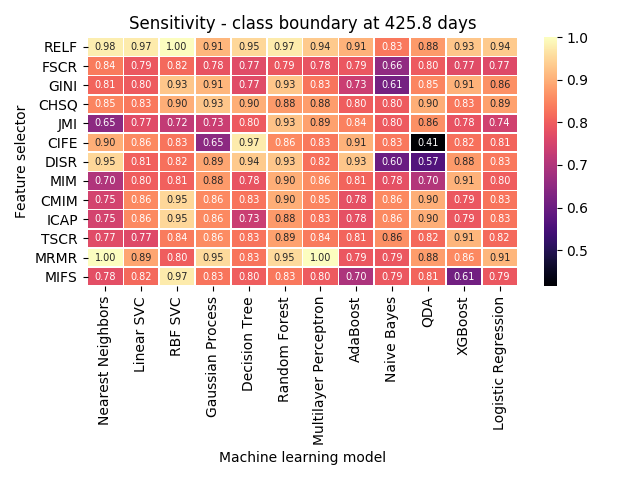

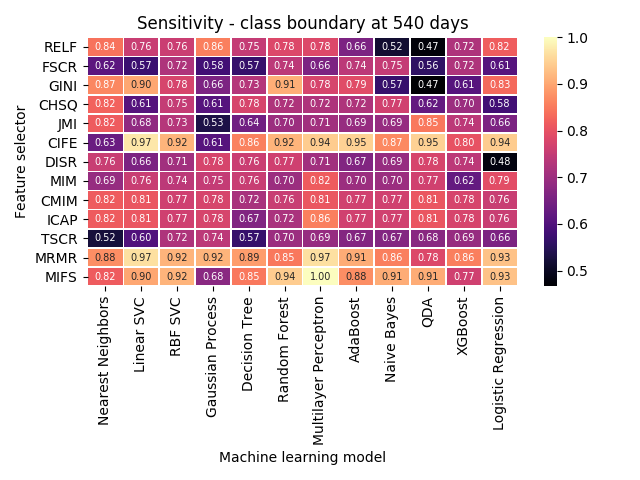

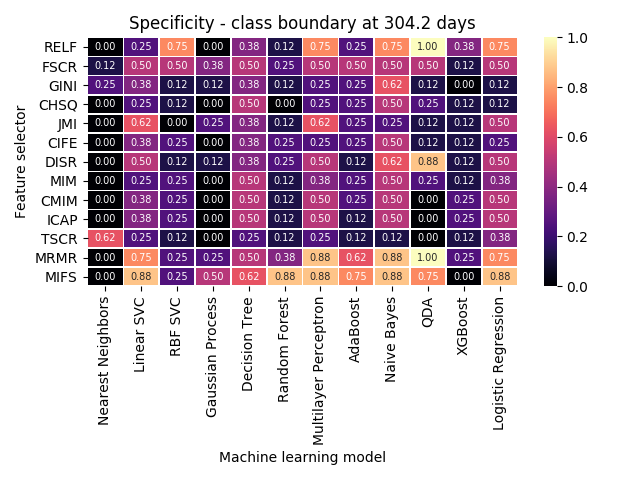

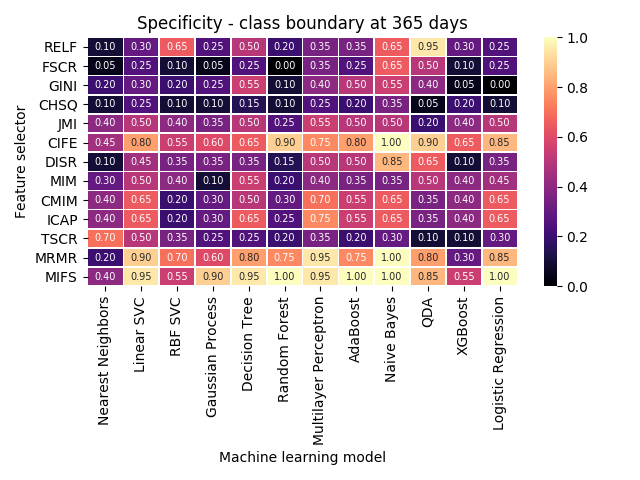

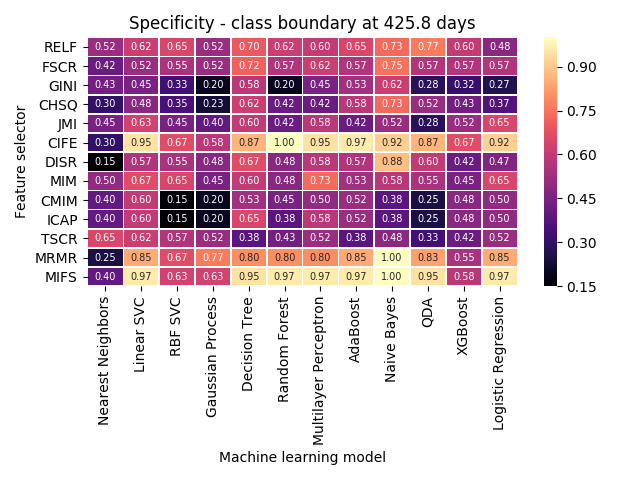

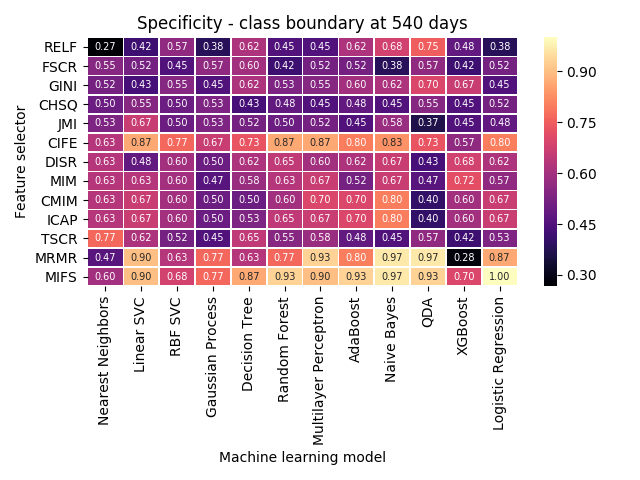


### Three Overall Survival Classes


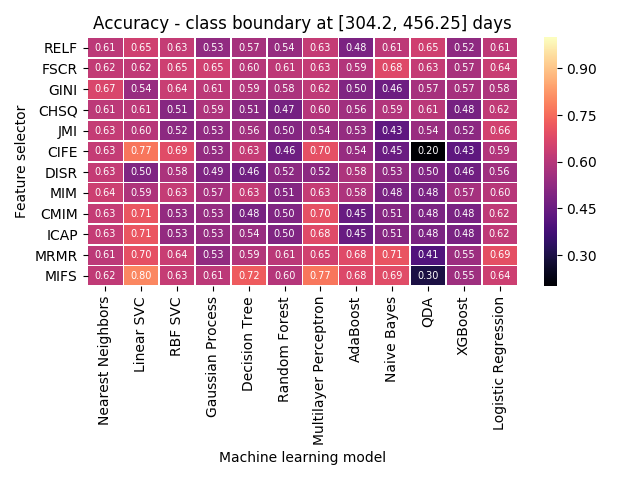

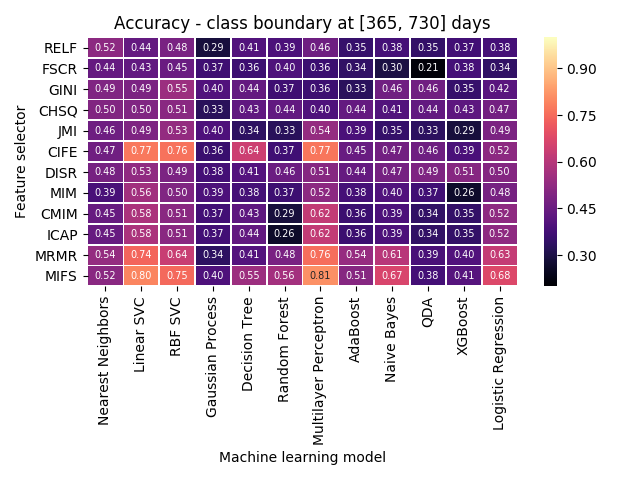

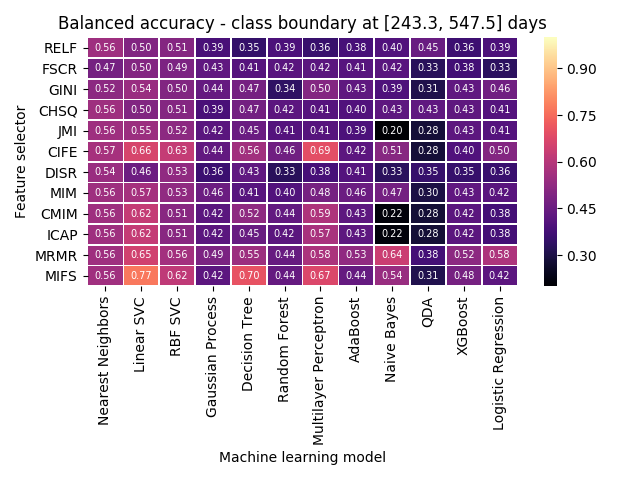

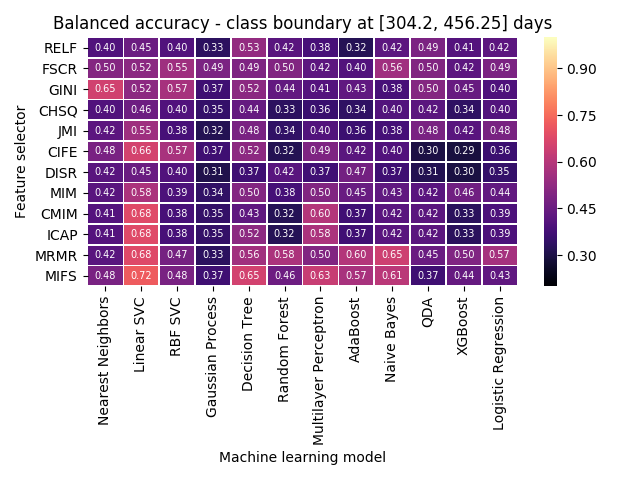

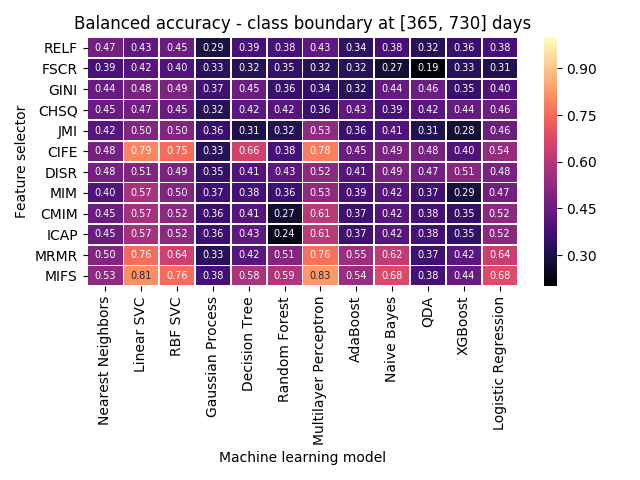

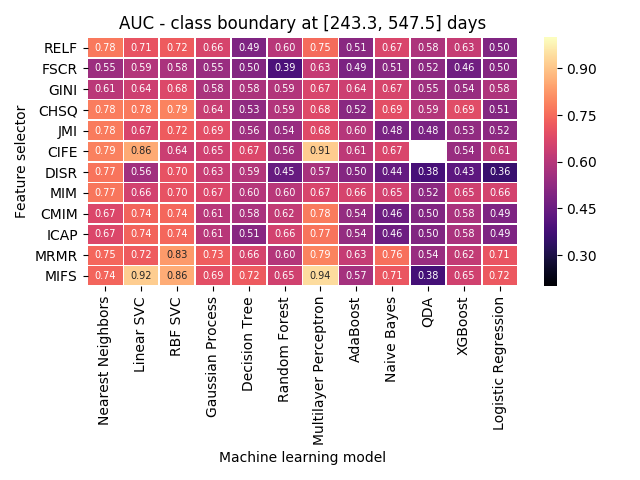

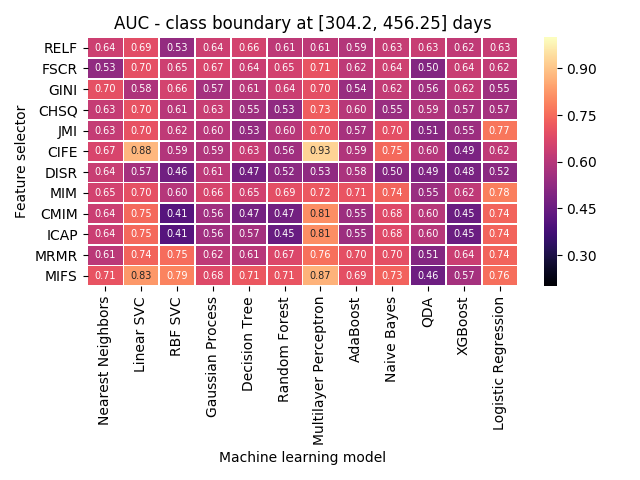

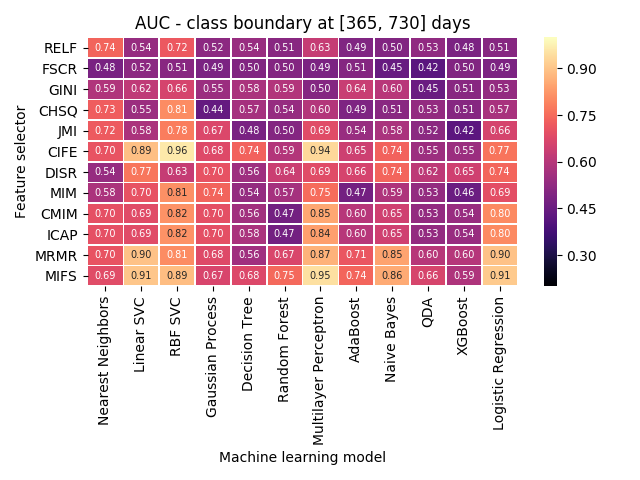

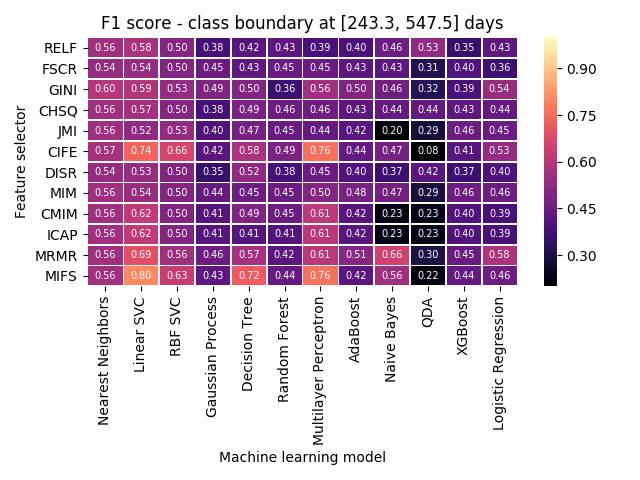

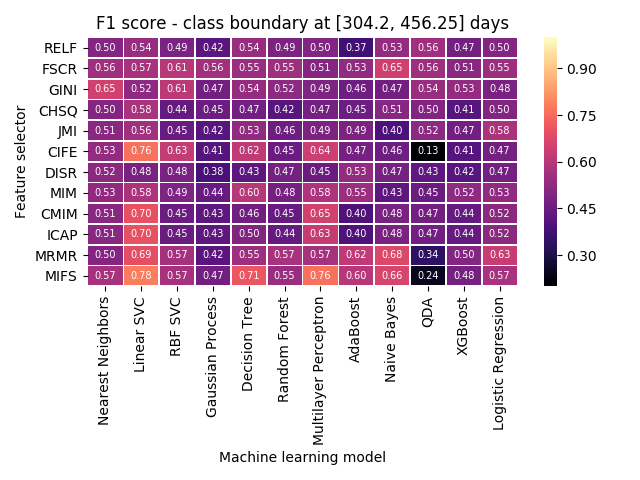

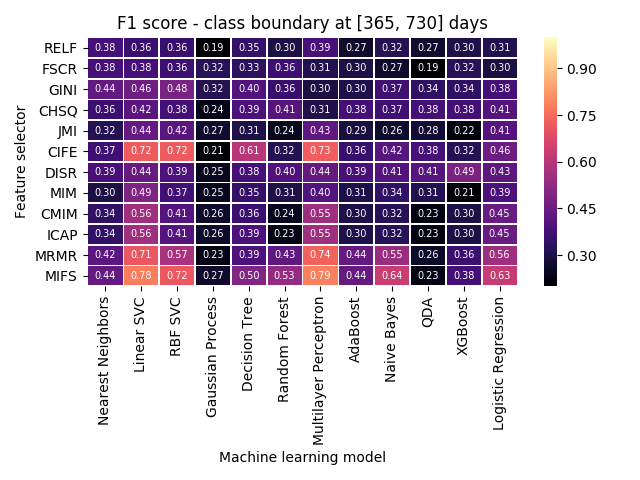

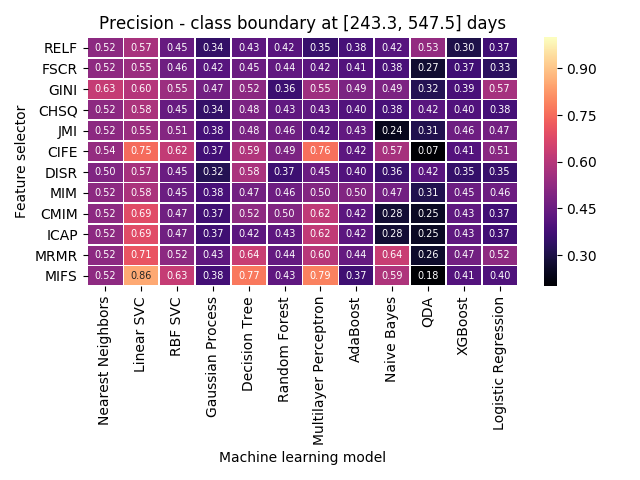

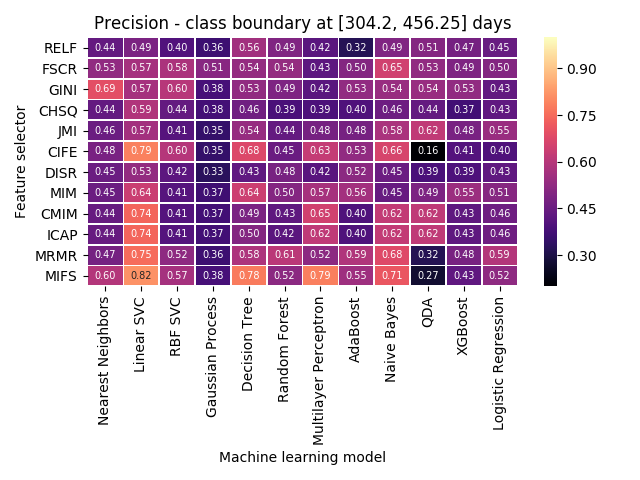

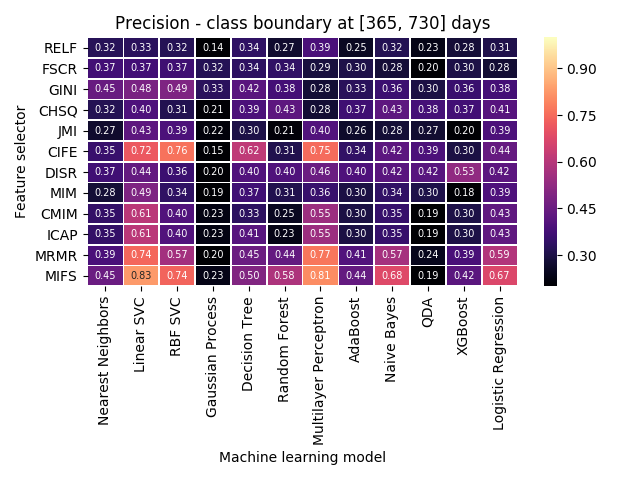

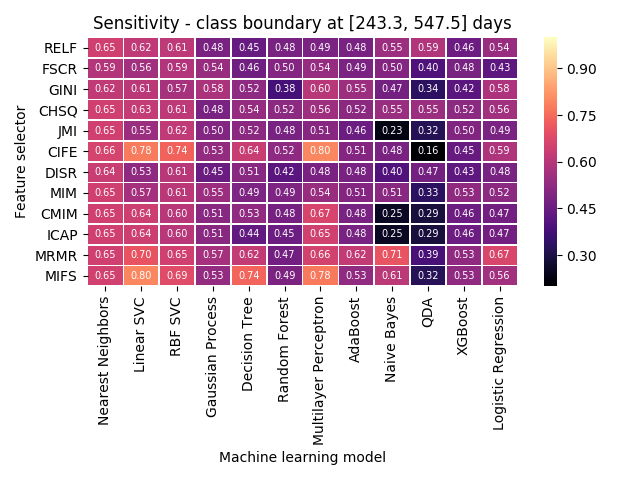

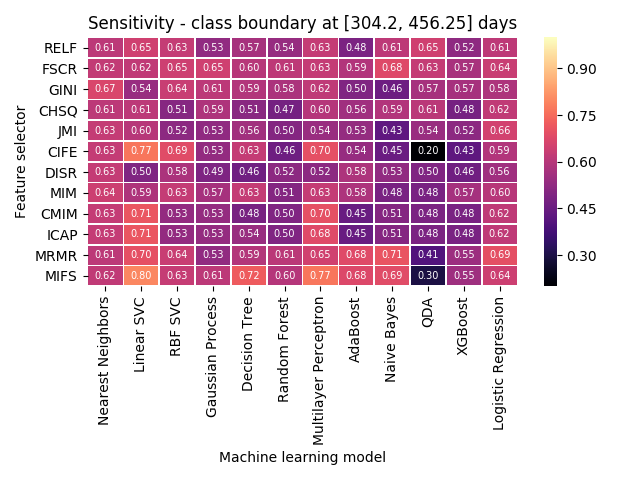

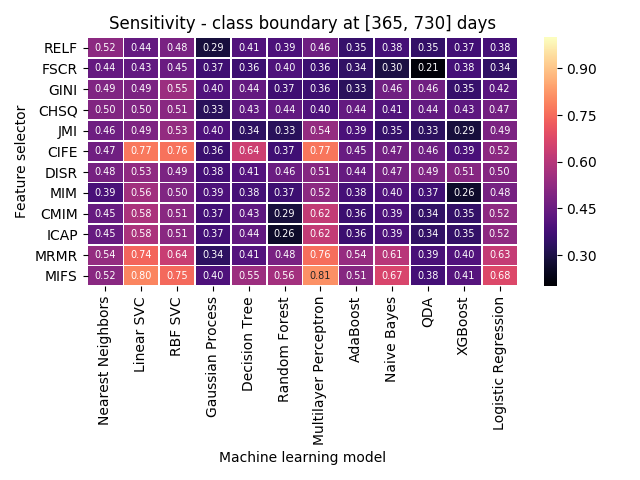

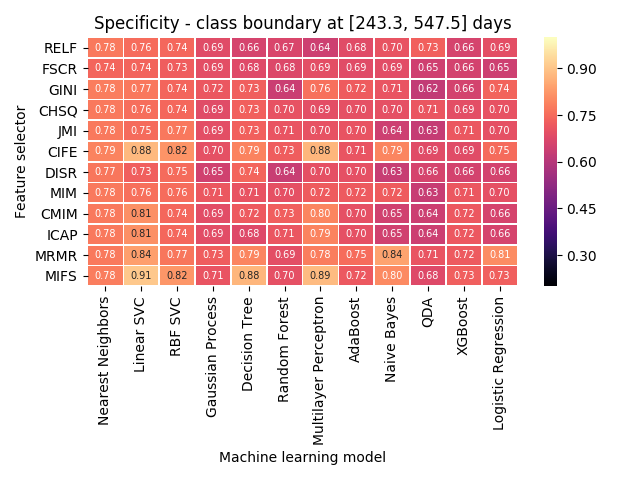

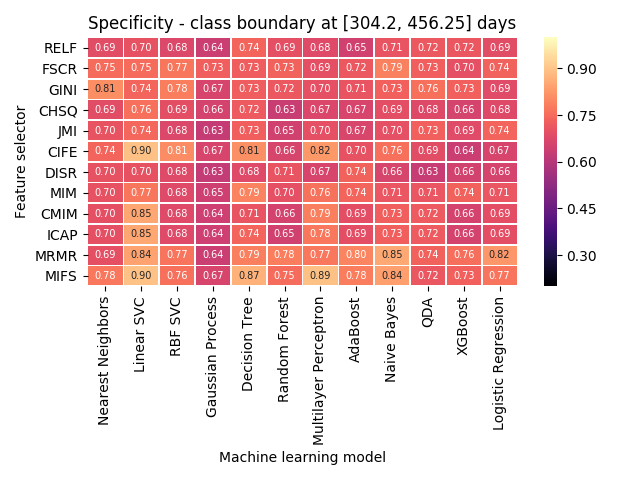

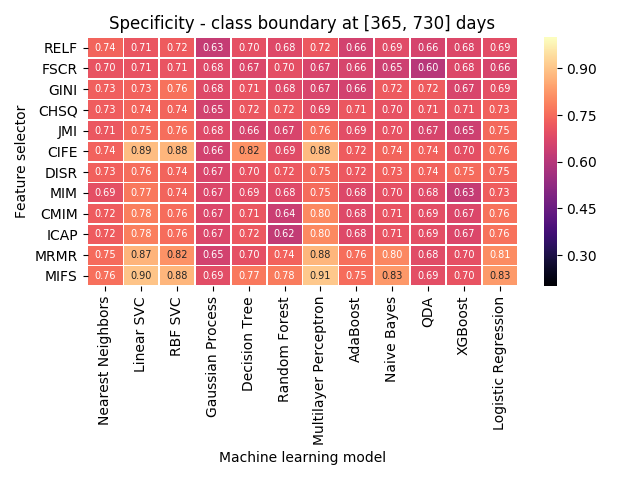

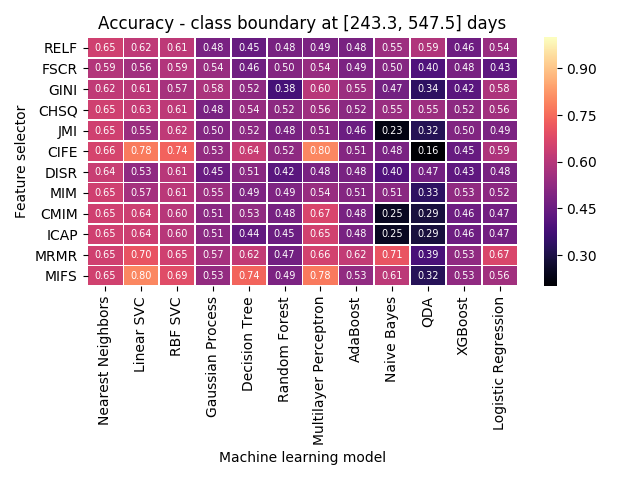


## S9 Performance on Multi-Center Data

Table S4 and Figure S7 contain the full performance metric comparison for all two and three OS class boundaries, while in the main document, only the balanced accuracy and AUC for the two OS class experiments were listed.

**Table S4**: Comparison of best-performing feature selectors (by AUC). Due to the high class imbalance for class boundaries at high and low overall survival values, the balanced accuracy is reported. The single-center metrics are listed as the mean across splits of stratified 10-fold cross-validation. A large performance drop can be observed when the model is tested on unseen multi-center data. Abbreviations: C: clinical prior, H: hand-picked, S: single-center, M: multi-center, bal. acc.: balanced accuracy, MRMR: minimum redundancy maximum relevance, MIFS: mutual information feature selection, CIFE: conditional infomax feature extraction, RELF: ReliefF, GINI: Gini index, CMIM: conditional mutual information maximization, MLP: multi-layer perceptron, RBF SVC: support vector classifier with radial basis function kernel.

|  | **Prior** | **Center** | **Robustness** | **Class Boundary** | **Selector** | **ML model** | **AUC** | **Bal. Acc** | **Acc** | **Spec** | **Sens** | **F1** | **Precision** |
| --- | --- | --- | --- | --- | --- | --- | --- | --- | --- | --- | --- | --- | --- |
| **Two overall survival classes** | - | S | Non-robust | 304.20 | MRMR | Gaussian Process | 1.00 | 70% | 91% | 25% | 100% | 0.95 | 91% |
|  | - | S | Non-robust | 365.00 | MIFS | MLP | 0.98 | 93% | 91% | 95% | 90% | 0.93 | 98% |
|  | - | S | Non-robust | 425.80 | CIFE | Adaboost | 1.00 | 94% | 93% | 97% | 91% | 0.94 | 98% |
|  | - | S | Non-robust | 540.00 | MRMR | MLP | 1.00 | 95% | 95% | 93% | 97% | 0.95 | 95% |
|  | - | M | Non-robust | 304.20 | MRMR | Gaussian Process | 0.50 | 52% | 61% | 6% | 98% | 0.75 | 60% |
|  | - | M | Non-robust | 365.00 | MIFS | MLP | 0.42 | 50% | 47% | 8% | 92% | 0.62 | 47% |
|  | - | M | Non-robust | 425.80 | CIFE | AdaBoost | 0.49 | 51% | 42% | 15% | 86% | 0.53 | 38% |
|  | - | M | Non-robust | 540.00 | MRMR | MLP | 0.51 | 55% | 43% | 30% | 80% | 0.43 | 29% |
|  | C | S | Robust | 304.20 | RELF | Random Forest | 0.76 | 69% | 90% | 25% | 98% | 0.94 | 91% |
|  | C | S | Robust | 365.00 | RELF | Nearest Neighbors | 0.72 | 52% | 73% | 10% | 94% | 0.84 | 77% |
|  | C | S | Robust | 425.80 | RELF | XGBoost | 0.81 | 71% | 75% | 55% | 86% | 0.83 | 81% |
|  | C | S | Robust | 540.00 | GINI | Decision Tree | 0.69 | 68% | 68% | 70% | 67% | 0.69 | 78% |
|  | C | M | Robust | 304.20 | RELF | Random Forest | 0.54 | 50% | 59% | 0% | 100% | 0.74 | 59% |
|  | C | M | Robust | 365.00 | RELF | Nearest Neighbors | 0.57 | 51% | 49% | 13% | 89% | 0.62 | 48% |
|  | C | M | Robust | 425.80 | RELF | XGBoost | 0.49 | 45% | 41% | 28% | 62% | 0.44 | 35% |
|  | C | M | Robust | 540.00 | GINI | Decision Tree | 0.46 | 43% | 45% | 46% | 40% | 0.28 | 21% |
|  | H | S | Robust | 304.20 | CIFE | XGBoost | 0.90 | 75% | 93% | 38% | 100% | 0.96 | 93% |
|  | H | S | Robust | 365.00 | MRMR | AdaBoost | 0.78 | 69% | 77% | 50% | 88% | 0.85 | 85% |
|  | H | S | Robust | 425.80 | MRMR | AdaBoost | 0.82 | 67% | 72% | 52% | 83% | 0.79 | 79% |
|  | H | S | Robust | 540.00 | GINI | AdaBoost | 0.74 | 68% | 69% | 53% | 82% | 0.73 | 71% |
|  | H | M | Robust | 304.20 | CIFE | XGBoost | 0.66 | 57% | 64% | 16% | 98% | 0.77 | 63% |
|  | H | M | Robust | 365.00 | MRMR | AdaBoost | 0.54 | 58% | 57% | 30% | 86% | 0.65 | 53% |
|  | H | M | Robust | 425.80 | MRMR | AdaBoost | 0.51 | 50% | 45% | 28% | 72% | 0.50 | 38% |
|  | H | M | Robust | 540.00 | GINI | Adaboost | 0.48 | 43% | 42% | 41% | 45% | 0.29 | 21% |
| **Three overall survival classes** | - | S | Non-Robust | 243.3 \| 547.5 | MIFS | MLP | 0.94 | 67% | 78% | 89% | 78% | 0.76 | 79% |
|  | - | S | Non-Robust | 304.2 \| 456.25 | CIFE | MLP | 0.93 | 49% | 70% | 83% | 70% | 0.64 | 63% |
|  | - | S | Non-Robust | 365 \| 730 | CIFE | RBF SVC | 0.96 | 75% | 76% | 88% | 76% | 0.72 | 76% |
|  | - | M | Non-Robust | 243.3 \| 547.5 | MIFS | MLP | 0.49 | 30% | 26% | 66% | 26% | 0.19 | 22% |
|  | - | M | Non-Robust | 304.2 \| 456.25 | CIFE | MLP | 0.47 | 28% | 28% | 65% | 28% | 0.22 | 42% |
|  | - | M | Non-Robust | 365 \| 730 | CIFE | RBF SVC | 0.60 | 49% | 38% | 72% | 38% | 0.32 | 48% |
|  | C | S | Robust | 243.3 \| 547.5 | RELF | RBF SVC | 0.68 | 49% | 60% | 74% | 60% | 0.53 | 52% |
|  | C | S | Robust | 304.2 \| 456.25 | RELF | Gaussian Process | 0.73 | 35% | 58% | 66% | 58% | 0.46 | 39% |
|  | C | S | Robust | 365 \| 730 | CMIM | RBF SVC | 0.65 | 48% | 53% | 75% | 53% | 0.50 | 50% |
|  | C | M | Robust | 243.3 \| 547.5 | RELF | RBF SVC | 0.58 | 30% | 28% | 66% | 28% | 0.23 | 26% |
|  | C | M | Robust | 304.2 \| 456.25 | RELF | Gaussian Process | 0.49 | 35% | 34% | 67% | 34% | 0.19 | 37% |
|  | C | M | Robust | 365 \| 730 | CMIM | RBF SVC | 0.48 | 35% | 25% | 66% | 25% | 0.23 | 37% |
|  | H | S | Robust | 243.3 \| 547.5 | GINI | RBF SVC | 0.74 | 60% | 64% | 78% | 64% | 0.61 | 61% |
|  | H | S | Robust | 304.2 \| 456.25 | CIFE | XGBoost | 0.74 | 42% | 53% | 71% | 53% | 0.50 | 52% |
|  | H | S | Robust | 365 \| 730 | GINI | RBF SVC | 0.64 | 45% | 45% | 71% | 45% | 0.41 | 42% |
|  | H | M | Robust | 243.3 \| 547.5 | GINI | RBF SVC | 0.58 | 34% | 38% | 67% | 38% | 0.29 | 25% |
|  | H | M | Robust | 304.2 \| 456.25 | CIFE | XGBoost | 0.47 | 33% | 33% | 67% | 33% | 0.17 | 11% |
|  | H | M | Robust | 365 \| 730 | GINI | RBF SVC | 0.49 | 33% | 16% | 67% | 16% | 0.04 | 2% |


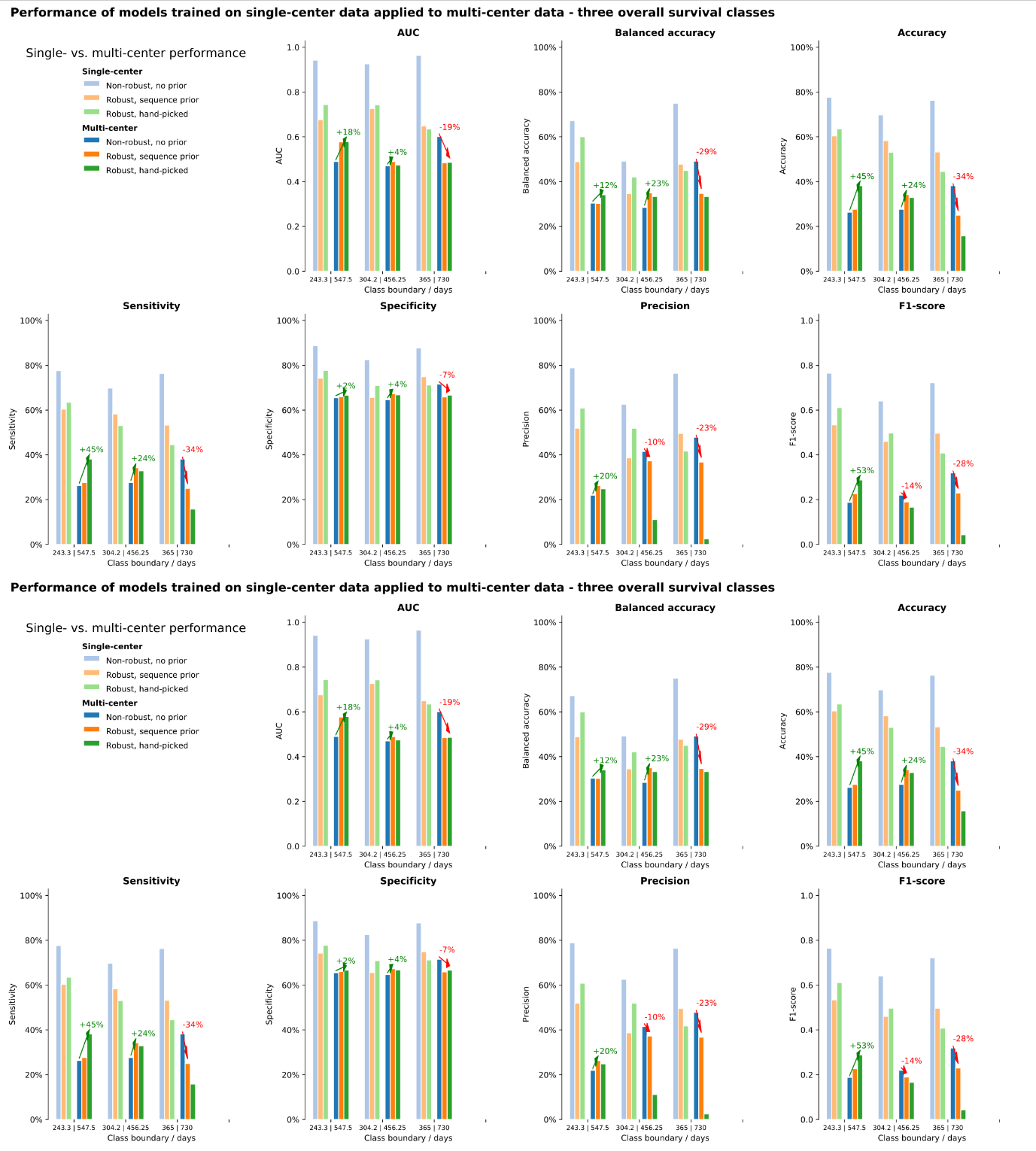


**Figure S7** Performance comparison single- versus multi-center for two and three overall survival classes. Shown for non-robust feature sets, robust features with clinical prior, and hand-picked feature selection. The results show the trade-off between single-center performance and the drop when moving to multi-center data. Introducing priors helped reduce performance drop. The arrows indicate whether a prior increased performance on multi-center data when compared to the non-robust features. The benefit of robust features highly depends on the class boundary used, since different feature selections and machine learning methods were used.

**Table S5**: Acquisition parameters retrieved from the DICOM information. All values are shown as mean ± standard deviation.

| **Dataset** | **Sequence** | **Field strength  / T** | **Rows** | **Columns** | **Slices** | **Thickness  / mm** | **Slice spacing  / mm** | **In-plane voxel size / mm** | | **Echo time  / ms** | **Repetition time  / ms** | **Inversion time  /ms** | **Flip angle /°** | **Specific absorption rate** |
| --- | --- | --- | --- | --- | --- | --- | --- | --- | --- | --- | --- | --- | --- | --- |
| Single-center | T1c | 1.93±0.69 | 274±67 | 271±58 | 162±16 | 1.00±0.02 | 0.05±0.22 | 0.97±0.12 x | 0.97±0.12 | 4.34±1.05 | 1896.00±374.52 | 1029.27±114.55 | 13.64±3.76 | 0.04±0.05 |
| BraTS-TCIA | T1c | 1.73±0.54 | 290±77 | 193±27 | 83±77 | 3.49±1.71 | 4.41±2.34 | 0.81±0.19 x | 0.81±0.19 | 8.01±4.35 | 576.00±629.24 | 414.00±497.43 | 64.67±30.29 | 0.31±0.56 |
| Single-center | T1 | 1.93±0.69 | 337±91 | 309±76 | 73±66 | 3.55±1.93 | 3.86±2.92 | 0.75±0.22 x | 0.75±0.22 | 5.85±3.82 | 945.63±667.36 | 993.75±112.36 | 57.64±35.48 | 0.36±0.35 |
| BraTS-TCIA | T1 | 1.73±0.54 | 288±78 | 173±39 | 53±68 | 4.34±1.10 | 5.00±1.75 | 0.82±0.16 x | 0.82±0.16 | 8.17±4.77 | 536.64±610.55 | 381.36±471.34 | 77.40±23.57 | 0.30±0.58 |
| Single-center | T2 | 1.93±0.69 | 391±119 | 368±108 | 103±73 | 2.88±1.98 | 2.78±2.98 | 0.69±0.25 x | 0.69±0.25 | 246.52±148.97 | 3705.31±715.08 | - | 131.55±15.53 | 0.27±0.21 |
| BraTS-TCIA | T2 | 1.73±0.54 | 322±104 | 217±49 | 56±43 | 4.10±1.49 | 4.74±2.23 | 0.73±0.24 x | 0.73±0.24 | 91.78±27.04 | 3775.32±1057.80 | - | 106.15±32.25 | 0.33±0.56 |
| Single-center | Flair | 1.93±0.69 | 410±111 | 354±105 | 42±23 | 3.67±0.69 | 4.42±0.81 | 0.60±0.22 x | 0.60±0.22 | 95.52±38.56 | 8498.57±842.73 | 2454.98±123.83 | 153.43±17.65 | 0.28±0.22 |
| BraTS-TCIA | Flair | 1.73±0.54 | 287±77 | 181±35 | 35±26 | 4.81±0.62 | 5.44±1.48 | 0.77±0.17 x | 0.77±0.17 | 131.42±18.75 | 9500.69±735.43 | 2257.20±123.70 | 111.92±35.70 | 0.17±0.24 |

# References

1. van Griethuysen JJM, Fedorov A, Parmar C, et al. Computational Radiomics System to Decode the Radiographic Phenotype. *Cancer Res*. 2017;77(21):e104-e107. doi:10.1158/0008-5472.CAN-17-0339

2. Lowekamp BC, Chen DT, Ibáñez L, Blezek D. The Design of SimpleITK. *Front Neuroinform*. 2013;7:45. doi:10.3389/fninf.2013.00045

3. Li J, Cheng K, Wang S, et al. Feature Selection. *ACM Comput Surv*. 2017;50(6):1-45. doi:10.1145/3136625

4. Pedregosa F, Varoquaux G, Gramfort A, et al. Scikit-learn: Machine Learning in Python. *J Mach Learn Res*. 2011;12(Oct):2825-2830. http://jmlr.csail.mit.edu/papers/v12/pedregosa11a.html. Accessed December 18, 2019.

5. Porz N, Habegger S, Meier R, et al. Fully Automated Enhanced Tumor Compartmentalization: Man vs. Machine Reloaded. *PLoS One*. 2016. doi:10.1371/journal.pone.0165302

6. Meier R, Knecht U, Loosli T, et al. Clinical Evaluation of a Fully-automatic Segmentation Method for Longitudinal Brain Tumor Volumetry. *Sci Rep*. 2016;6(1):23376. doi:10.1038/srep23376

7. Tustison NJ, Avants BB, Cook PA, et al. N4ITK: improved N3 bias correction. *IEEE Trans Med Imaging*. 2010;29(6):1310-1320. doi:10.1109/TMI.2010.2046908

8. Reuter M, Rosas HD, Fischl B. Highly accurate inverse consistent registration: A robust approach. *Neuroimage*. 2010;53(4):1181-1196. doi:10.1016/J.NEUROIMAGE.2010.07.020

9. Robnik-Šikonja M, Kononenko I. Theoretical and Empirical Analysis of ReliefF and RReliefF. *Mach Learn*. 2003;53(1/2):23-69. doi:10.1023/A:1025667309714

10. Duda RO, Hart PE (Peter E, Stork DG. *Pattern Classification*. Wiley; 2001. https://dl.acm.org/citation.cfm?id=954544. Accessed December 18, 2019.

11. Gini C. Variabilità e mutabilità (Variability and Mutability). *Repr Mem di Metodol Stat (Ed Pizetti E, Salvemini, T) Rome Libr Eredi Virgilio Veschi ed Bol*. 1912.

12. Huan Liu, Setiono R. Chi2: feature selection and discretization of numeric attributes. In: *Proceedings of 7th IEEE International Conference on Tools with Artificial Intelligence*. IEEE Comput. Soc. Press; :388-391. doi:10.1109/TAI.1995.479783

13. Yang HH, Moody JE. Data Visualization and Feature Selection: New Algorithms for Nongaussian Data. *undefined*. 1999. https://www.semanticscholar.org/paper/Data-Visualization-and-Feature-Selection%3A-New-for-Yang-Moody/6980ab0f9f460d6cf2acaddeb1388524b0f481b8. Accessed December 18, 2019.

14. Richa R, Sznitman R, Taylor R, Hager G. Visual tracking using the sum of conditional variance. *2011 IEEE/RSJ Int Conf Intell Robot Syst*. 2011;(2):2953-2958. doi:10.1109/IROS.2011.6094650

15. Meyer PE, Schretter C, Bontempi G. Information-Theoretic Feature Selection in Microarray Data Using Variable Complementarity. *IEEE J Sel Top Signal Process*. 2008;2(3):261-274. doi:10.1109/JSTSP.2008.923858

16. Lewis DD, D. D. Feature selection and feature extraction for text categorization. In: *Proceedings of the Workshop on Speech and Natural Language - HLT ’91*. Morristown, NJ, USA: Association for Computational Linguistics; 1992:212. doi:10.3115/1075527.1075574

17. Fleuret F. Fast Binary Feature Selection with Conditional Mutual Information. *J Mach Learn Res*. 2004;5(Nov):1531-1555. http://www.jmlr.org/papers/v5/fleuret04a.html. Accessed December 18, 2019.

18. Akadi A El, El Akadi A, El Ouardighi A, Aboutajdine D. A powerful feature selection approach based on mutual information. *Int J Comput Sci Netw Secur*. 2008:116--121. http://citeseerx.ist.psu.edu/viewdoc/summary?doi=10.1.1.522.6487. Accessed December 18, 2019.

19. Davis JC, C. J. *Statistics and Data Analysis in Geology*. Wiley; 1986. https://dl.acm.org/citation.cfm?id=533006. Accessed December 18, 2019.

20. Hanchuan Peng, Fuhui Long, Ding C. Feature selection based on mutual information criteria of max-dependency, max-relevance, and min-redundancy. *IEEE Trans Pattern Anal Mach Intell*. 2005;27(8):1226-1238. doi:10.1109/TPAMI.2005.159

21. Battiti R. Using mutual information for selecting features in supervised neural net learning. *IEEE Trans Neural Networks*. 1994;5(4):537-550. doi:10.1109/72.298224

22. Cover T, Hart P. Nearest neighbor pattern classification. *IEEE Trans Inf Theory*. 1967;13(1):21-27. doi:10.1109/TIT.1967.1053964

23. Platt JC, Platt JC. Probabilistic Outputs for Support Vector Machines and Comparisons to Regularized Likelihood Methods. *Adv LARGE MARGIN Classif*. 1999:61--74. http://citeseer.ist.psu.edu/viewdoc/summary?doi=10.1.1.41.1639. Accessed December 18, 2019.

24. Rasmussen CE, Williams CKI. *Gaussian Processes for Machine Learning*. MIT Press; 2006. http://www.gaussianprocess.org/gpml/. Accessed December 18, 2019.

25. Gordon AD, Breiman L, Friedman JH, Olshen RA, Stone CJ. Classification and Regression Trees. *Biometrics*. 1984;40(3):874. doi:10.2307/2530946

26. Breiman L. Random Forests. *Mach Learn*. 2001;45(1):5-32. doi:10.1023/A:1010933404324

27. Hinton GE. Connectionist Learning Procedures. *Mach Learn*. January 1990:555-610. doi:10.1016/B978-0-08-051055-2.50029-8

28. Hastie T, Rosset S, Zhu J, Zou H. Multi-class AdaBoost. *Stat Interface*. 2009;2(3):349-360. doi:10.4310/SII.2009.v2.n3.a8

29. Freund Y, Schapire RE. A Decision-Theoretic Generalization of On-Line Learning and an Application to Boosting. *J Comput Syst Sci*. 1997;55(1):119-139. doi:10.1006/JCSS.1997.1504

30. Chan TF, Golub GH, LeVeque RJ. Updating Formulae and a Pairwise Algorithm for Computing Sample Variances. In: *COMPSTAT 1982 5th Symposium Held at Toulouse 1982*. Heidelberg: Physica-Verlag HD; 1982:30-41. doi:10.1007/978-3-642-51461-6_3

31. Ledoit O, Wolf M. Honey, I Shrunk the Sample Covariance Matrix. *J Portf Manag*. 2004;30(4):110-119. doi:10.3905/jpm.2004.110

32. Chen T, Guestrin C. XGBoost: A Scalable Tree Boosting System. March 2016. doi:10.1145/2939672.2939785

33. Verhulst PF. La loi d’accroissement de la population. 1845.
